# Multi‐Crystal X‐Ray Diffraction (MCXRD) Bridges the Crystallographic Characterisation Gap in Chemistry and Materials Science: Application to MOFs

**DOI:** 10.1002/anie.202523233

**Published:** 2026-01-18

**Authors:** Joshua P. Smith, Rebecca Smith, Thomas M. Roseveare, Dominic Bara, Alexander J. R. Thom, Ross S. Forgan, Mark R. Warren, Anna J. Warren, Robin L. Owen, Lee Brammer

**Affiliations:** ^1^ Chemistry Division MPS School University of Sheffield Brook Hill Sheffield S3 7HF UK; ^2^ Diamond Light Source Harwell Science and Innovation Campus Didcot OX11 0DE UK; ^3^ School of Chemistry University of Glasgow Joseph Black Building, University Avenue Glasgow G12 8QQ UK

**Keywords:** Big data, Crystal structure, Metal‐organic framework, Radiation damage, Serial crystallography

## Abstract

Structure determination by X‐ray diffraction is limited by crystal size and can be compromised by radiation damage when using very intense X‐ray radiation. X‐ray structure determination from partial diffraction data sets combined from multiple crystals is a potential solution, but its exploitation in chemistry and materials science is largely unrealized. Here we report the use of synchrotron radiation for multi‐crystal X‐ray diffraction (MCXRD) adapted for structure determination of metal‐organic framework (MOF) materials with crystal dimensions too small for conventional single‐crystal diffraction studies. We further show that radiation‐induced chemical changes and degradation of diffraction quality can be alleviated. Our approach encompasses both rotation‐ and stationary‐MCXRD measurements for 10 to 1000s of crystals with software‐optimized combination of the multiple data sets. We report the crystal structures of six MOFs: MOF‐919(Sc/Cu), MET‐2, MIL‐88B(Cr)‐1,4‐NDC, PCN‐260(Sc), UiO‐66, and UiO‐66‐MoO_4_ with unit cell dimensions ranging from 18−114 Å and crystal sizes from 0.5−480 µm^3^. This approach can address the challenges of structure determination in a regime of particle size and sample radiation sensitivity that lies between existing single‐crystal X‐ray diffraction and the emerging field of electron diffraction. MCXRD can provide accurate atomic‐resolution structure determination for some of the most challenging cases in chemistry and materials science.

## Graphical abstract

Multi‐crystal X‐ray diffraction (MCXRD) methods allow accurate structural characterisation with crystals too small for single‐crystal X‐ray diffraction and too large for electron diffraction, as demonstrated here for metal‐organic frameworks, and alleviate the radiation‐induced chemical changes that can result from using high‐intensity X‐ray sources to study very small crystals.

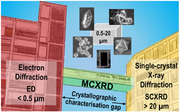

## Introduction

Single‐crystal X‐ray diffraction (SCXRD) is regarded as the gold standard for structural characterization in chemistry and materials science as it provides an atomic‐resolution description of molecular and crystal structures, enabling a platform for developing and understanding structure‐property relationships. There remain classes of materials, often at the forefront of research areas, however, for which such structure determination remains either highly challenging or even elusive. Major impediments include the inability to obtain large enough single crystals for diffraction experiments or the degradation of crystals by radiation damage from use of very intense X‐ray radiation sources to compensate for small crystal size. A number of solutions have been developed, including novel approaches to crystal growth^[^ ^1^, ^2^ ^]^ or periodic encapsulation of molecular compounds in framework materials (the “crystalline sponge” approach),^[^ ^3^, ^4^ ^]^ but these do not provide a universal solution. Powder X‐ray diffraction (PXRD) can in some cases enable crystal structure determination for crystalline materials with micron‐sized particles.^[^ ^5^ ^]^ The compression of 3D reciprocal space into a 1D diffractogram presents challenges of peak overlap, however, often preventing ab initio structure determination by PXRD. Knowledge of the approximate molecular or network structure can enable its refinement from PXRD data by Rietveld methods,^[^ ^6^ ^]^ although using a restricted structural model compared to the norms of SCXRD.

Dedicated chemical crystallography beamlines at synchrotrons have advanced the opportunities for SCXRD structure determination with smaller crystals,^[^ ^7^ ^]^ although with challenges due to radiation damage from use of intense X‐ray beams focussed on the smallest crystals. Global radiation damage causes diffraction intensities to diminish during data collection, starting with high‐angle reflections, reducing data completeness and overall data set resolution.^[^ ^8^ ^]^ Site‐specific radiation damage is more insidious and proceeds at much faster rates than for global damage, leading to site‐specific chemical changes in situ, often well before degradation of the diffraction pattern becomes obvious.^[^ ^9^ ^]^ Consequently, in structure determination, interpreting, and modelling electron density distributions becomes more challenging and is often accompanied by higher *R*‐values, error accumulation in refined atomic displacement parameters (ADPs) and model‐derived bond lengths deviating from expected values.^[^ ^8^ ^]^ Most recently, electron diffraction (ED) has emerged as an exciting prospect for crystal structure determination as the strong Coulomb interaction of crystals with the incident electron beam, compared to the interaction of X‐rays, generates Bragg reflections with higher signal‐to‐noise ratios for smaller (50–500 nm size) crystals.^[^ ^10^, ^11^ ^]^ The technique has evolved rapidly from development of experimental methods to measurement of diffraction data in an automated manner^[^ ^12^ ^]^ and the advent of commercial instrumentation. Challenges still remain, however, with refinement of crystal structure models, due to the need to account for dynamical scattering, which is prominent in electron diffraction, rather than kinematic scattering which describes X‐ray diffraction.

Metal‐organic frameworks (MOFs) are a class of materials that present many of the greatest challenges, but also opportunities, for structure determination by diffraction methods. These chemically and structurally diverse crystalline materials with molecular‐scale pores have attracted widespread interest and research activities across science and engineering disciplines for applications including molecular sensing, drug delivery, catalysis, and gas or liquid separations.^[^ ^13^, ^14^, ^15^, ^16^ ^]^ Structure determination is a pre‐requisite to understanding a material's properties and the development of applications. Finding suitable crystallization conditions to enable SCXRD can, however, be elusive and require a time‐consuming trial‐and‐error process, especially when first exploring unknown chemical space in the pursuit of novel framework materials.  This is particularly true for high‐valent MOFs, which are the subject of considerable attention due to their resistance to chemical degradation resulting from strong metal‐ligand bonds.^[^ ^17^, ^18^ ^]^ Lability and reversibility of metal‐ligand bond formation, which best facilitates larger crystal growth via supramolecular assembly, is consequently restricted, ensuring micro‐crystalline powders dominate the crystallization landscape for such MOFs. This often prevents crystal structure determination or requires synchrotron PXRD and Rietveld refinement methods to establish, at best, a highly constrained structural model.^[^ ^19^ ^]^ The Cambridge Structural Database (CSD) includes 1715 3D MOF structures determined by PXRD.^[^ ^20^ ^]^ These are important but, for reasons already noted, have limitations on crystal structure model accuracy compared to SCXRD studies and may cause subtle aspects of molecular dynamics and crystallographic disorder to be overlooked.^[^ ^19^ ^]^

Many of the challenges outlined above are also prevalent in macromolecular crystallography where the difficulties in obtaining large, well‐diffracting crystals led to the development of microfocus beamlines.^[^ ^21^ ^]^ Here, the beam size can be matched to small crystal sizes (<10 µm), which increases the signal‐to‐noise, improving the data quality, and potentially increasing the resolution of the data. The resulting increase in flux densities combined with the sensitivity of protein crystals to beam‐induced damage,^[^ ^9^ ^]^ however, necessitated the development, and wide uptake, of multi‐crystal approaches as a single crystal is no longer sufficient to obtain a complete, high‐resolution data set.^[^ ^22^ ^]^ MCXRD data collection, now well established in macromolecular crystallography, uses semi‐automated approaches for the sequential collection and processing of small wedges of diffraction data from a population of randomly orientated crystals.^[^ ^22^, ^23^ ^]^

Multi‐crystal approaches merge and scale together partial data sets from many crystals, typically a single wedge of reciprocal space from each crystal, all of which contribute to the completeness and quality of a single composite data set. The impact of radiation damage is minimized by dividing the X‐ray dose required for structure solution over many crystals with an angular range for each wedge chosen to avoid reaching the radiation‐dose limit for each crystal. The most extreme multi‐crystal approaches are classified as serial crystallography (SX). Driven initially by the demands of X‐ray free‐electron lasers (XFELs), where a new sample must be presented to each X‐ray pulse, SX approaches have become increasingly used to study biological macromolecules at synchrotrons (serial synchrotron crystallography, SSX) in recent years, including for time‐resolved studies.^[^ ^24^ ^]^ Serial crystallography typically entails the collection of a single static diffraction image from each crystal, using specialized sample delivery approaches,^[^ ^25^ ^]^ although the SX moniker is sometimes relaxed to include data collection from very small angular wedges (< 5°).^[^ ^26^ ^]^

**Figure 1:**
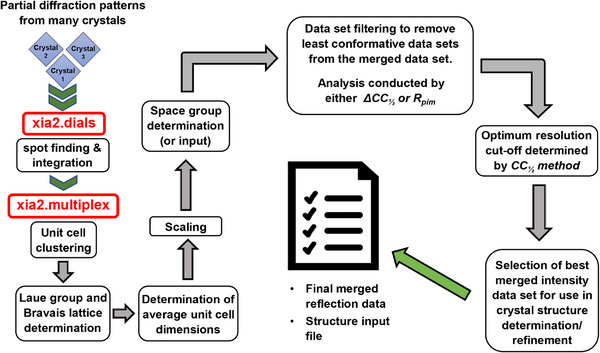
X‐Ray data processing pipeline. Partial X‐ray data sets from 10s of crystals (< 120) for rotation‐MCXRD to 1000s of crystals for stationary‐MCXRD (SX) were classified for data quality, indexed, selectively combined (i.e., outlier removal), and used for crystal structure solution and refinement. The automated processing pipeline developed for this purpose to harness a sequence of existing software is illustrated here for use with rotation‐MCXRD data. The approach used for stationary‐MCXRD (SX) using *xia2.ssx* is described in the Supporting Information.

**Figure 2:**
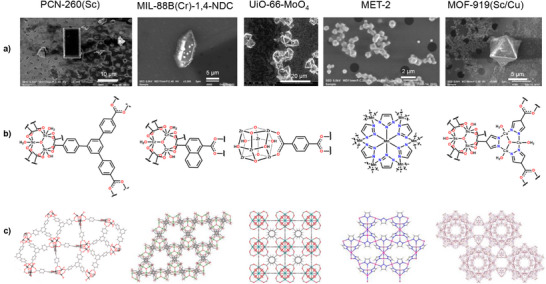
a) SEM images of crystals for PCN‐260(Sc), MIL‐88B(Cr)‐1,4‐NDC, UiO‐66‐MoO_4_, MET‐2, and MOF‐919(Sc/Cu), showing crystal morphology and size. Crystals of UiO‐66 (prior to post‐synthetic modification) were the same size as for UiO‐66‐MoO_4_. b) Building blocks, and c) crystal structures of the MOFs PCN‐260(Sc), MIL‐88B(Cr)‐1,4‐NDC, UiO‐66, MET‐2, and MOF‐919(Sc/Cu). Building blocks (secondary building units, SBUs) are presented with only one of the linker ligands shown in full. The crystal structure of UiO‐66‐MoO_4_ has the same network as UiO‐66 and is presented in more detail in Figure 4. PCN‐260(Sc) = [Sc_3_(μ_3_‐O)(OH)(OH_2_)_2_(BTB)_2_], BTB = benzene‐1,3,5‐tribenzoate; MIL‐88B(Cr)‐1,4‐NDC = [Cr_3_(μ_3_‐O)(OH)(OH_2_)_2_(1,4‐NDC)_3_], NDC = naphthalenedicarboxylate; UiO‐66 = [Zr_6_(μ_3_‐O)_4_(μ_3_‐OH)_4_(1,4‐BDC)_6_], BDC = benzenedicarboxylate; MET‐2 = [Mn(C_2_H_2_N_3_)_2_]; MOF‐919(Sc/Cu) = [{Sc_3_(μ_3_‐O)(OH)_3_}(μ‐PyC)_6_{Cu_3_(μ_3_‐O)(OH_2_)_3_}_2_], and H_2_PyC = 4‐pyrazolecarboxylic acid.

These multi‐crystal approaches, however, have seen only limited development for applications in chemistry and materials science,^[^ ^27^, ^28^, ^29^, ^30^, ^31^, ^32^ ^]^ where the problems of radiation damage^[^ ^8^ ^]^ and structure determination from very small crystals have only more recently come to the forefront. In this study we demonstrate the efficacy of MCXRD approaches, including SX, to such problems (Figure 1) and have focussed on MOFs, which represent the range of challenges to be overcome in the more difficult structure determination cases within these disciplines. We selected six MOFs for structure determination by these methods: PCN‐260(Sc), MIL‐88B(Cr)‐1,4‐NDC, UiO‐66, UiO‐66‐MoO_4_, MET‐2, and MOF‐919(Sc/Cu) (Figure 2). These choices complement studies reported for MIL‐100 and ZIF‐8.^[^ ^31^ ^]^ For each material in the present study, it is either very difficult or currently not possible to obtain crystals large enough for conventional SCXRD studies or such studies have only yielded structure refinements of limited quality. These MOFs have unit cell dimensions ranging from 18−114 Å with average crystal sizes from 0.5−480 µm^3^ (Figure 2a). The upper end of this crystal size range may be viable for SCXRD at a suitable synchrotron beamline, if radiation damage permits, whereas the lower end of the range overlaps with crystal sizes being used for ED. Therefore, the chosen systems traverse the gap in crystallographic characterization between conventional SCXRD studies and emerging ED structure determination. At the same time, they represent a gradation in the challenge for MCXRD structure determination and have allowed development and validation of the approaches. They collectively illustrate the effectiveness and future breadth of application of the multi‐crystal and serial crystallography to MOFs and more widely in chemistry and materials science.

## Results and Discussion

The MOFs MOF‐919(Sc/Cu),^[^ ^33^ ^]^ MET‐2,^[^ ^34^ ^]^ and UiO‐66^[^ ^35^ ^]^ were synthesized according to reported procedures, yielding crystals of comparable size to those originally reported, wherein structure determination was originally restricted to PXRD studies. UiO‐66‐MoO_4_ was prepared by post‐synthetic modification of UiO‐66 with retention of crystal size, wherein catalytic molybdate sites were introduced by reaction with Na_2_MoO_4_. Synthesis and structural characterization of UiO‐66‐MoO_4_, along with PCN‐260(Sc), and MIL‐88B(Cr)‐1,4‐NDC has not previously been reported. Collectively these materials have allowed us to develop MCXRD/SX approaches to crystal structure determination by single‐crystal methods that overcome the challenges related to small crystal size, weak diffraction, and/or susceptibility to radiation damage associated with these materials (Figures 1 and 2). MCXRD data were collected by either rotation or stationary‐crystal (SX) approaches at beamlines I04,^[^ ^36^ ^]^ I24,^[^ ^37^ ^]^ or VMXm,^[^ ^38^ ^]^ Diamond Light Source, using high‐intensity focussed X‐ray beams optimized in full‐width half‐maximum (FWHM) to the crystal size used. For sample delivery during data collection, the crystals were distributed across either a Mylar film (I04, I24) or a cryo‐electron microscopy (cryoEM) grid (VMXm) and measurements were made at room temperature other than for MOF‐919(Sc/Cu).

### The Data Analysis Pipeline: Generating a Single Merged Data Set for Crystal Structure Determination

An automated processing pipeline has been used for crystal structure determination of samples pertinent to chemistry and materials science by MCXRD methods and has been applied to the six MOF crystal structures reported herein. The pipeline developed for use in rotation‐MCXRD studies is shown in Figure 1, whereas for stationary‐MCXRD (SX) an established pipeline that employs the *xia2.ssx* ^[^ ^39^ ^]^ software was used. These pipelines enable identification of diffracting crystals, indexing of their diffraction patterns and combination of partial data sets with optimized outlier removal, leading to a final merged X‐ray data set from which crystal structure determination and refinement can be undertaken. For rotation‐MCXRD studies, two data set filtering approaches were compared to yield the best combined data set for use in structure determination. Both approaches harness the *xia2.dials* ^[^ ^40^ ^]^ and *xia2.multiplex* ^[^ ^23^ ^]^ software, developed for automation of macromolecular crystallography, including MCXRD, to direct data reduction using the *DIALS* software.^[^ ^41^, ^42^ ^]^ These software modules were coupled with locally written scripts to enable automation of the pipeline in the manner described herein (Figure 1). One approach involved filtering of the partial data sets (Tables S22–S27) based on a recursive application of the Δ*CC*_1/2_ method^[^ ^43^, ^44^ ^]^ to merged data sets from which the least conformative data sets were removed.^[^ ^45^ ^]^ The second involved screening based upon a suitable measure of agreement (*R*_pim_)^[^ ^46^, ^47^ ^]^ between redundant data, enabling sequential removal of the lowest quality partial data set. The best combined data set was then selected based upon a compromise of data completeness, mean *I*/σ(*I*) and overall agreement between redundant data (*R*_pim_). The resolution cut‐off for each combined data set was determined by the *CC*_1/2_ method^[^ ^44^ ^]^ and indicated that a reduction from the maximum accessible resolution was needed only for PCN‐260(Sc) and MOF‐919(Sc/Cu). The best determined combined data set for each material was then used for crystal structure determination by conventional SCXRD methods. A full summary of the data collected and methodology used to obtain the final combined data set for both rotation‐MCXRD and stationary‐MCXRD (SX) studies is provided in the Supporting Information.

The MCXRD approaches allowed the crystal structures of the six selected MOFs to be solved and refined by conventional SCXRD crystallographic methods, using *SHELX*,^[^ ^48^ ^]^ as implemented via *OLEX2*.^[^ ^49^ ^]^ MCXRD data quality enabled refinement of most non‐hydrogen atoms with anisotropic displacement parameters. Some least‐squares parameter constraints or restraints were required and the contribution to the diffraction intensities of highly disordered pore solvent was accounted for in the final stages of refinement by solvent masking algorithms, as is common in crystal structures of MOFs. Crystallographic data and crystal structure refinement parameters are provided in Table S1 and crystal structures are shown in Figure 2.

### Developing the MCXRD Methodology: Crystal Structures of PCN‐260(Sc) and MIL‐88B(Cr)‐1,4‐NDC

**Figure 3:**
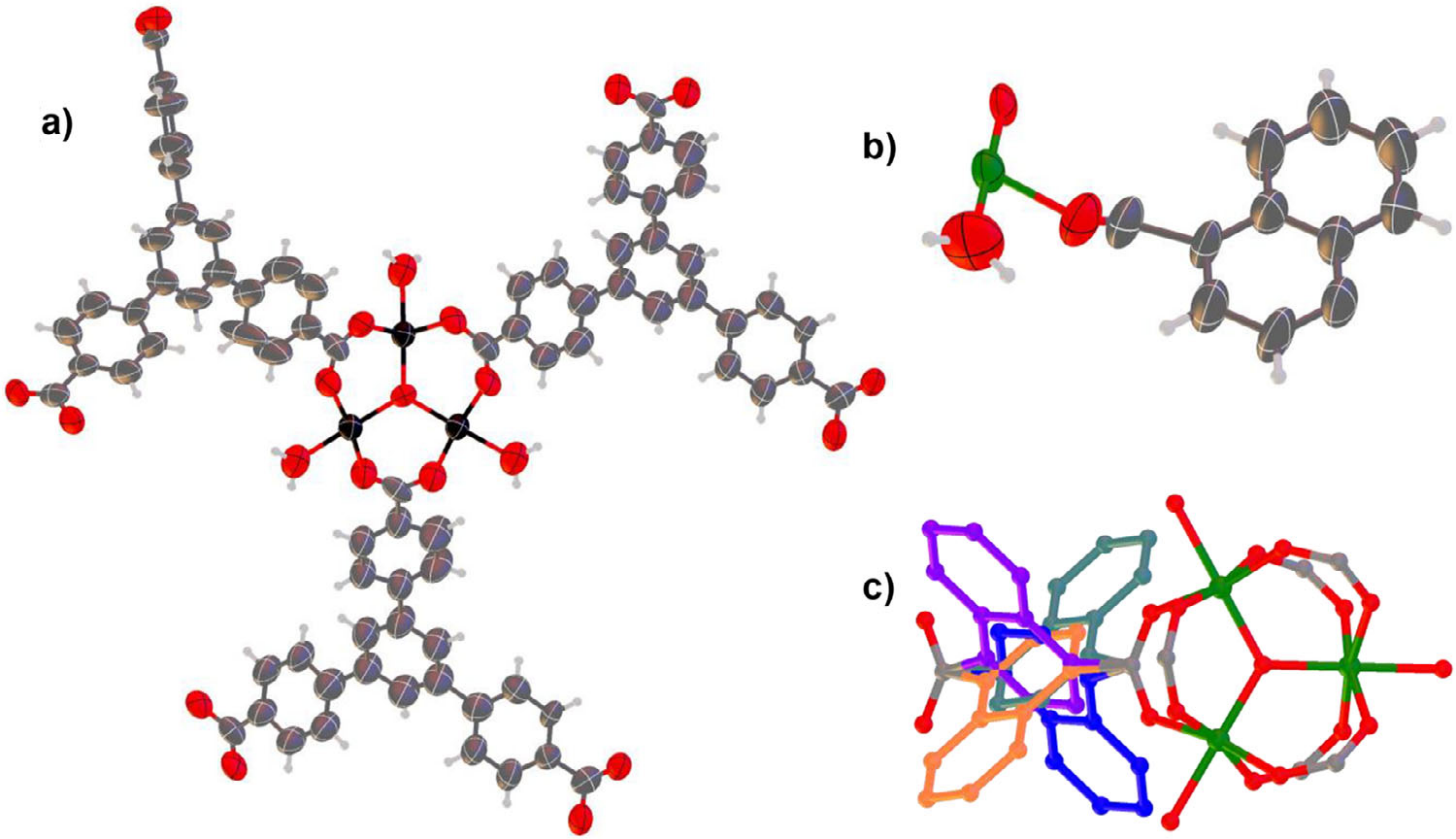
Structure detail for PCN‐260(Sc) and MIL‐88(Cr)‐1,4‐NDC. a) Connectivity of Sc_3_(μ_3_‐O)(OH)(OH_2_)_2_ building block in PCN‐260(Sc), shown with three of the six connecting BTB ligands. b) Asymmetric unit of MIL‐88(Cr)‐1,4‐NDC. c) Four‐fold rotational disorder of 1,4‐NDC ligand attached to Cr_3_(μ_3_‐O)(OH)(OH_2_)_2_ building block of MIL‐88(Cr)‐1,4‐NDC with each 1,4‐NDC ligand orientation shown in a different colour. Sc atoms, black; Cr atoms, green; C atoms, grey; O atoms, red. Anisotropic displacement ellipsoids are represented at the 50% level for non‐hydrogen atoms in (a) and (b).

PCN‐260(Sc) yielded crystals with maximum dimensions of 10–20 µm, which are at the lower end of crystals suitable for SCXRD crystal structure determination from individual crystals using synchrotron radiation, assuming sufficient stability to radiation damage. Although the crystal structure of the PCN‐260(Fe_2_Co) analogue has previously been determined from large single crystals (300–500 µm),^[^ ^50^ ^]^ the scandium analogue has not previously been reported and yields much smaller crystals. These crystals provide a good test of the MCXRD methodology and allowed the initial optimisation of the approach from one designed for macromolecular crystallography to one that can be effective for chemical and materials crystallography, where smaller unit cell sizes provide greater challenges in accumulating sufficient data for indexing on each separate crystal. X‐Ray intensity data were collected as angular wedges, using exposure times of 0.05 s per 0.1° frame, for 54 crystals of which 23 yielded indexed diffraction data (Tables S2, S8, S9, and S22). Many data sets had poor intensity statistics (e.g., *R*_pim_ > 0.2). Data merged from only 4 crystals (*R*_pim_ < 0.13) provided the best combined data set for crystal structure solution and refinement for PCN‐260(Sc). This outcome reflects the challenge of the low diffraction quality present in the many rejected data sets, one of the common difficulties faced in structural characterisation of poorly crystalline materials, including many of the more challenging MOF materials, but also demonstrates the advantage of the relatively large angular wedges that were accessible for these crystals. The crystal structure (orthorhombic, *Pca*2_1_, *a* = 36.23930(13) Å, *b* = 18.7201(2) Å, and *c* = 49.2460(4) Å) comprises four independent benzenetribenzoate ligands that adopt twisted conformations to link the Sc_3_(μ_3_‐O)(O_2_CR)_6_ secondary building units (SBUs) shown in Figure 3a and is consistent with the PCN‐260(Fe_2_Co) analogue.^[^ ^50^ ^]^

Direct synthesis of MIL‐88B(Cr)‐1,4‐NDC yielded crystals of dimensions 3–15 µm, smaller than those of PCN‐260(Sc), but presenting a substantial challenge for structural characterization. Homonuclear Cr(III) MOFs are particularly chemically and thermally stable but also have proven largely elusive for SCXRD structure determination^[^ ^51^ ^]^ due to the very small crystal size that is accessible with the kinetically inert Cr(III) ions, which limit reversibility of metal‐ligand bond formation during supramolecular assembly needed to promote MOF crystal growth.^[^ ^2^, ^18^, ^52^ ^]^ To our knowledge there are no reported SCXRD studies of such MOFs prepared by direct synthesis rather than post‐synthetic metal‐ion exchange and none of the highly flexible MIL‐88(Cr) family of MOFs^[^ ^53^ ^]^ has been previously characterized by SCXRD. Following an analogous experimental approach to that used for PCN‐260(Sc), rotation‐MCXRD diffraction data collected from 19 crystals at room temperature were processed via the filtering pipeline to provide a complete data set for structure determination derived from scaling and merging data retained from 6 crystals (Tables S3, S10, S11, and S23). The crystal structure (hexagonal, *P*6_3_/*mmc*, *a* = 14.1170(4) Å, *c* = 17.2012(5) Å) comprises Cr_3_(μ_3_‐O)(O_2_CR)_6_ SBUs linked via 1,4‐naphthalenedicarboxylate (1,4‐NDC) ligands, which display a turnstile‐like 4‐fold rotational disorder in contrast to the Fe_2_Co analogue known as PCN‐241.^[^ ^50^ ^]^ The asymmetric unit of the crystal structure is shown in Figure 3b and the 4‐fold ligand disorder is illustrated in Figure 3c. MIL‐88B(Cr)‐1,4‐NDC is a member of an archetypal family of MOFs (MIL‐88) that undergo a very large continuous swelling behavior in response to guest content over an unusually large dynamic range.^[^ ^53^ ^]^ The crystal structure reported herein lies midway between the most open and most closed forms of the MOF, and has unit cell dimensions consistent with those determined by PXRD for the DMF‐solvated form (see Figure S1).

### Application in Catalytic Site Identification: 5 µm Crystals of UiO‐66 and UiO‐66‐MoO_4_

**Figure 4:**
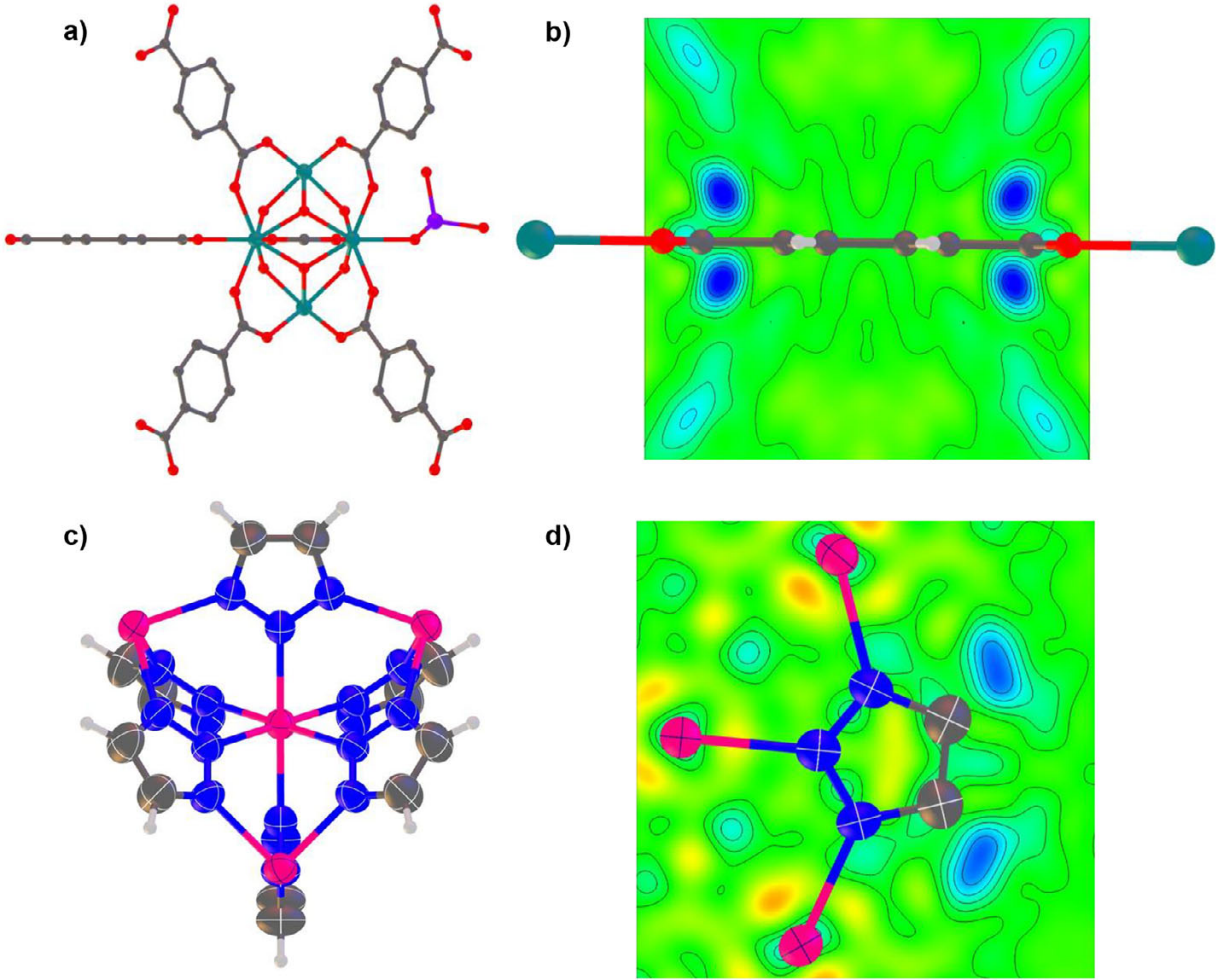
Structure detail for UiO‐66‐MoO_4_ and MET‐2. a, b) Crystal structure of UiO‐66‐MoO_4_ showing a) coordination of molybdate to an edge site of a Zr_6_ network node (SBU), replacing a terephthalate linker ligand, and b) residual electron density in absence of molybdate modelling, indicating 4‐fold disorder of molybdate site (contours at 0.25 e Å^−3^). c, d) Crystal structure of MET‐2 showing c) coordination environment of Mn(II) sites (atoms shown with 50% atomic displacement ellipsoids), and d) electron density difference map illustrating direct location of hydrogen atoms (contours at 0.084 e Å^−3^).

UiO‐66 synthesis^[^ ^54^, ^55^ ^]^ yielded crystals of 5 µm in diameter, which were post‐synthetically modified by reaction with Na_2_[MoO_4_] to incorporate the molybdate ion (MoO_4_^2−^) as a site for oxidation catalysis (UiO‐66‐MoO_4_). Such crystals are smaller than can typically be used for crystal structure determination from one crystal although larger crystals of UiO‐66 have been amenable to SCXRD structure determination.^[^ ^55^ ^]^ For UiO‐66, data from 50 crystals led to selection of 10 data sets from the processing pipeline to merge into the combined data set (Tables S4, S12, S13, and S24) used for crystal structure determination and anisotropic refinement of all non‐hydrogen atoms (Figure S2), yielding a final structure consistent with that reported from larger single crystals.^[^ ^55^ ^]^ For UiO‐66‐MoO_4_ partial data sets from 76 crystals resulted in a final merged data set from 20 crystals (Tables S5, S14, S15, and S25). Structure solution and refinement of the initial framework model enabled identification of residual electron density above and below the ligand plane, consistent with μ‐κ^1^,κ^1^‐MoO_4_^2−^ coordination at an edge site of the Zr_6_ octahedron of the SBU (Figures 4a, S3). Unlike a previous study in which molybdate has been reported tethered to Zr_6_ octahedra of a larger‐pore MOF,^[^ ^56^ ^]^ the structure determination of UiO‐66‐MoO_4_ presents a particular challenge due to the high symmetry of the site and because the molybdate anion replaces only some of the terephthalate linkers at the catalytic loading used (Figure 4b). Refinement of the molybdate unit as a rigid body enabled chemically sensible location and an overall composition [Zr_6_(O)_4_(OH)_4_(1,4‐BDC)_6‐x_(MoO_4_)_x_], *x* = 1.14(7), to be determined, which compares well with ICP‐MS data that indicate *x* = 1.64. A full report of the catalytic behaviour of this material will be presented in a subsequent publication.

### Application to Sub‐Micron Crystals: Crystal Structure of MET‐2

The synthesis of MET‐2 yielded crystals with a size distribution of 0.7–1 µm (Figure 2a), which are substantially smaller than accessible for SCXRD structure determination from one crystal. Structure determination was originally reported from PXRD data by use of the charge‐flipping method.^[^ ^34^ ^]^ The material is one of a family of small‐pore MOFs (MET family; MET = Metal Triazolate) with surface areas comparable to zeolites. Rotation‐MCXRD data were collected on 71 crystals at beamline VMXm leading to a final combined data set from 25 crystals (Tables S6, S16, S17, and S26). Crystal structure solution allowed full anisotropic refinement to *R*1 = 0.031 including location of hydrogen atoms of the triazole ligands from difference electron density maps (Figure 4c, d) and their refinement without positional constraints or restraints.

### Very Large Unit Cell and Severe Radiation Sensitivity: Crystal Structure of MOF‐919(Sc/Cu)

**Figure 5:**
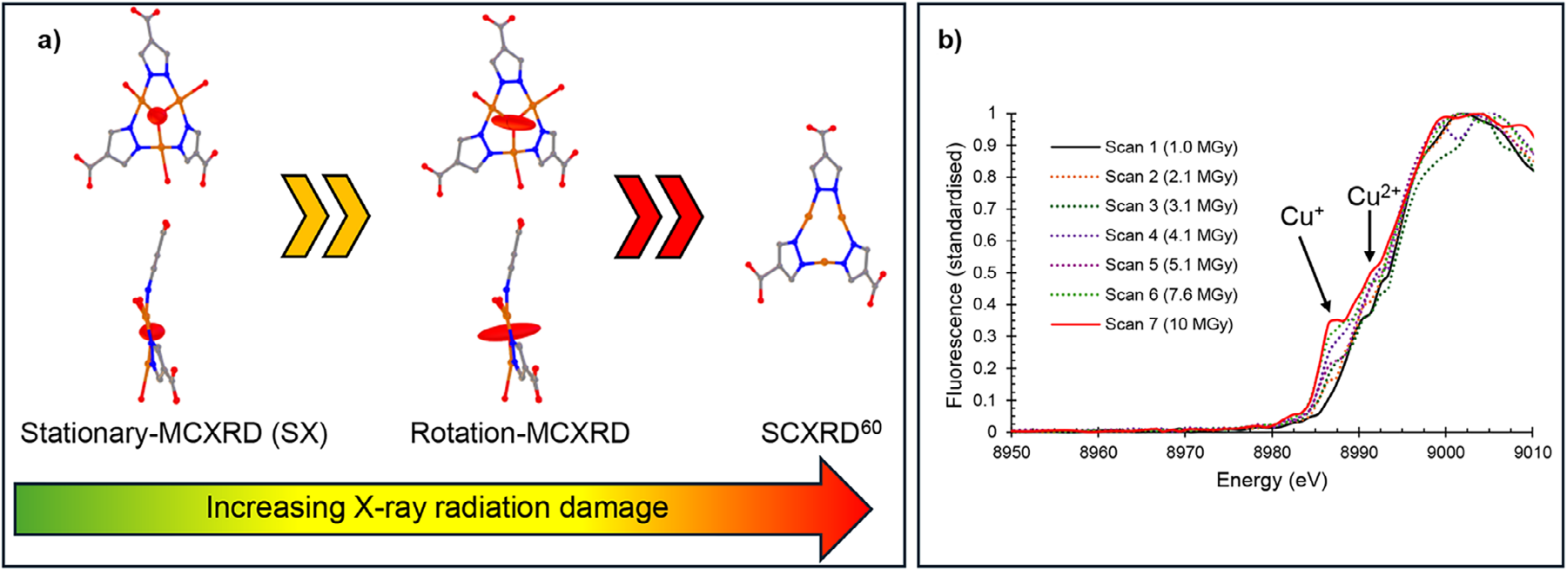
Crystal structures and effects of radiation damage for MOF‐919(Sc/Cu). a) Effects of global and site‐specific radiation damage are manifested in increased atomic displacement ellipsoids for μ_3_‐O site of the Cu_3_L_3_ SBU (L = 4‐carboxypyrazolate) and by progressive reduction of Cu(II) sites to Cu(I) leading finally to loss of oxide ligand. The greatest mitigation of radiation damage is achieved by use of stationary‐MCXRD (SX). b) X‐Ray fluorescence measurements correlate with X‐ray absorption and exhibit growth of a spectral feature upon exposure of crystals to increased X‐ray dose, evident from scan 2 onwards. This feature is attributed to Cu(I) sites (1s→4p absorption), confirming site‐specific radiation damage that results in Cu(II) to Cu(I) reduction. The cumulative average diffraction weighted dose (DWD)^[^ ^61^ ^]^ for scans 1–7 (1.00, 2.05, 3.06, 4.12, 5.12, 7.58, and 9.99 MGy, respectively) was calculated using RADDOSE‐3D.^[^ ^62^, ^63^ ^]^ See also Figure S4.

MOF‐919(Sc/Cu) forms crystals of 4–5 µm in size and is known from its original structural characterisation by PXRD to have unit cell dimensions of >100 Å,^[^ ^33^ ^]^ thereby representing an extremely challenging structure determination. Its large pores are capable of the inclusion of insulin^[^ ^33^ ^]^ and its Fe/Cu analogue has been shown to exhibit catalytic so‐called “nanozyme” behavior.^[^ ^57^ ^]^ The crystals also exhibit extreme radiation sensitivity, despite data collection at low temperature, which we attribute in part to site‐specific radiation damage involving the redox‐active SBUs (vide infra). This is exacerbated by the need for longer exposure times (1.5–3 s per 0.1° frame; average diffraction‐weighted dose (DWD) per frame ≈ 2–3 MGy) with these very small, weakly diffracting crystals. Global radiation damage is evident from the data collected by rotation‐MCXRD (117 crystals; retention and merging of 39 data sets; see Tables S7, S18, S19, and S27) by the rapid fall‐off in diffraction intensities (*I* and *I*/σ(*I*), Figure S4) and markedly worsening agreement of equivalent X‐ray reflection intensities as a function of X‐ray radiation dose. In order to further mitigate radiation damage by reducing X‐ray exposure of each crystal, single‐image data collection by stationary‐MCXRD (SX) at 3023 locations on a CryoEM grid was undertaken, leading to merging of data sets from 1763 crystals (Tables S20 and S21). This provided a marked improvement in diffraction quality for the final merged data set (Tables S18 and S20) and led to an improved crystal structure model, including site‐specific changes, which we have been able to attribute to reducing the effects of radiation damage. Most notably there was a dramatic reduction in the highly elongated anisotropic displacement ellipsoid for some μ_3_‐O sites of the Cu_3_(PyC)_3_ SBUs (PyC = 4‐carboxypyrazolate) (Figure 5a). Confirmation of a location of site‐specific radiation damage has been made from X‐ray fluorescence measurements that map X‐ray absorption at energies near the Cu absorption edge, indicating the growth of a spectral feature indicative of Cu(I) formation, which has resulted from X‐ray‐induced reduction of Cu(II) sites in the MOF (Figure 5b). This X‐ray induced reduction has been documented in other Cu‐containing materials^[^ ^58^ ^]^ and redox activity, more generally, is known for the Cu_3_(PyC)_3_ SBU.^[^ ^59^ ^]^

Further evidence of X‐ray induced chemical changes arose during our studies when larger crystals (30 µm) of MOF‐919(Sc/Cu) were reported,^[^ ^60^ ^]^ enabling a SCXRD crystal structure determination from synchrotron X‐ray data that showed limited diffraction to a resolution of *d*_min_ = 1.34 Å, compared with *d*_min_ = 1.15 Å for our MCXRD studies. The SCXRD model also has much higher residual indices, is more highly constrained and contains missing atoms and some non‐physical ligand geometries (see Figures S5 and S6).^[^ ^60^ ^]^ These SCXRD model deficiencies are perhaps unsurprising given that accumulated radiation dose can be assumed to be much higher for the reported SCXRD study than for our MCXRD/SX studies, with an anticipated greater radiation damage. Indeed, the absence of some central μ_3_‐O sites and ligated water molecules in the reported SCXRD structure model is consistent with extensive radiation damage leading to Cu(II) to Cu(I) reduction. The effects of increased radiation damage upon increased accumulated dose per crystal on going from stationary‐MCXRD (SX) to rotation‐MCXRD to SCXRD are summarized sequentially in Figure 5a and described in more detail in the Supporting Information (Sections S4 and S5). Thus, we have been able to demonstrate the effectiveness of rotation‐MCXRD and to a greater extent stationary‐MCXRD (SX) in mitigating radiation damage that has a marked effect on crystal structure determination when X‐rays serve as a non‐benign structural probe.

## Conclusions and Outlook

In this study we show that synchrotron MCXRD is a highly effective approach to address the crystal structure determination problem for microcrystal‐forming MOFs and the consequent radiation damage that occurs when X‐ray flux is increased to compensate for small crystal size or weak diffraction. We have demonstrated the effectiveness of both rotation‐MCXRD and stationary‐MCXRD (SX) as methodologies and illustrated this with crystal structure determination of six MOFs with unit cell dimensions in the range 18−114 Å and average crystal sizes from 0.5−480 µm^3^. Both approaches mitigate the effect of radiation damage compared to conventional SCXRD. The stationary‐crystal approach goes furthest in reducing radiation dose per crystal, whereas rotation‐MCXRD is more effective and requires fewer crystals to study materials for which radiation damage is inherently less extreme. The success of these studies and the range of both unit cell and crystal dimensions indicates that the methodology will be widely applicable in chemistry and materials science, in which there remain many areas where challenges in obtaining suitable single crystals remain a barrier to accurate structural characterization.

In future, it is anticipated that an increasing number of synchrotron beamlines will be able to offer a MCXRD/SX approach to crystallographic studies that can advance the chemical and materials sciences. Such studies provide a complementary approach to ED (50–500 nm) and conventional SCXRD (≥ 5 µm) based on crystal size and can offer an optimum solution for crystals in the 0.5–20 µm size regime. The approach sits alongside PXRD and serial femtosecond crystallography (SFX) but offers the most accessible route to overcome the challenges faced in accurate structure determination. The limitations in crystal structure determination by PXRD have already been noted (vide supra). SFX employs a similar approach to SSX, although crystal delivery methods can differ and hit‐rate is lower, requiring larger numbers of crystals for success. The greatest limitation of SFX, however, is the requirement of an X‐ray free‐electron laser facility (XFEL), of which there are far fewer than there are synchrotrons.

There is much scope for expansion of MCXRD/SX methods to broaden their application still further, not only to other MOFs but more widely across chemistry and materials science. Thus, with necessary hardware and accompanying software development, a more generalized serial crystallography approach to data collection and structure determination under variable temperature, variable pressure and/or non‐ambient gaseous atmospheres will be feasible. More generally, the problem of X‐ray radiation damage in crystal structure determination is not restricted to synchrotron X‐ray facilities. High‐flux laboratory rotating‐anode X‐ray sources, although having X‐ray flux a few orders of magnitude lower than 3^rd^ generation synchrotrons, still cause radiation damage due to longer exposure times at these X‐ray fluxes. Thus, adaption of data collection methodology to enable serial approaches would alleviate this problem in such home laboratory studies. Recent breakthroughs in AI‐assisted structure factor phase determination and the anticipated extension of such AI approaches^[^ ^64^ ^]^ are only likely to enhance the possibilities for multi‐crystal crystallography.

Finally, we anticipate that MCXRD/SX methods will provide a number of future opportunities not only for new types of investigations, but for investigations that are, in principle, feasible by existing methods, but are currently prohibitively time inefficient. We will report upon such opportunities in due course.

## Supporting Information

Supporting Information is available and contains further experimental details, including syntheses, methods, and information on individual crystallographic data sets. The experimentally determined crystal structures, including structure factors, have been deposited as CIFs with the CSD and can be freely accessed online via by the joint Cambridge Crystallographic Data Centre and Fachinformationszentrum Karlsruhe Access Structures service at https://www.ccdc.cam.ac.uk/structures/ (CSD deposition numbers 2487825, PCN‐260(Sc); 2487826, MIL‐88B(Cr)‐1,4‐NDC; 2487827, UiO‐66; 2487828, UiO‐66‐MoO_4_; 2487829, MET‐2; 2487830, MOF‐919(Sc/Cu) from rotation‐MCXRD and 2487831, MOF‐919(Sc/Cu) from stationary‐MCXRD (SX)). The authors have cited additional references within the Supporting Information.^[^ ^65^, ^66^, ^67^, ^68^, ^69^, ^70^, ^71^, ^72^, ^73^, ^74^, ^75^, ^76^, ^77^, ^78^ ^]^

## Author Contributions

J. P. S., R. S., D. B., and A. J. R. T. synthesized the MOFs. R. S., and A. J. W. collected X‐ray data and conducted preliminary data analyses and structure refinements on UiO‐66 and UiO‐66‐MoO_4_. J. P. S., T. M. R., R. L. O., and A. J. W. collected X‐ray data on all other MOFs. J.P.S. wrote scripts used to further automate the data processing pipeline and conducted data processing with support from R. L. O., M. R. W., and A. J. W. All crystal structure determinations and refinements were conducted by J. P. S. with support from T. M. R. and L. B. R. L. O. conducted X‐ray fluorescence spectroscopy measurements. R. S. F. supervised experimental work conducted at University of Glasgow. R. L. O., M. R. W., and A. J. W. supervised experimental work conducted at Diamond Light Source. L. B. conceived the idea, undertook initial planning in discussions with R. L. O., M. R. W., A. J. W., and R. S. F., and provided overall supervision of the project. L. B. and J. P. S. drafted the manuscript. All authors contributed to writing, editing, and/or approved the final version of the manuscript.

## Conflict of Interests

The authors declare no conflict of interest.

## Supporting information



Supporting Information

Supporting Information

## Data Availability

The data that support the findings of this study are available in the supplementary material of this article.
