# Supplementary material for: Multi‐Crystal X‐Ray Diffraction (MCXRD) Bridges the Crystallographic Characterisation Gap in Chemistry and Materials Science: Application to MOFs
Source: Angew Chem Int Ed Engl. 2026 Jan 18;65(9):e23233. doi: 10.1002/anie.202523233 (PMC12929940; doi:10.1002/anie.202523233)
Supplement: Supplementary file 1 — Supporting Information [file ANIE-65-e23233-s001.pdf]

Supporting Information

Multi-Crystal X-Ray Diffraction (MCXRD) Bridges the Crystallographic Characterisation Gap in Chemistry and Materials Science: Application to MOFs

Joshua P. Smith,[1,2] Rebecca Smith,[1] Thomas M. Roseveare,[1] Dominic Bara,[3] Alexander J. R. Thom,[3] Ross S. Forgan,[3] Mark R. Warren,[2] Anna J. Warren,[2] Robin L. Owen,[2] Lee Brammer[1]

[1] Chemistry Division, MPS School, University of Sheffield, Brook Hill, Sheffield S3 7HF, UK. [2] Diamond Light Source, Harwell Science and Innovation Campus, Didcot OX11 0DE, UK. [3] School of Chemistry, University of Glasgow, Joseph Black Building, University Avenue, Glasgow G12 8QQ, UK.

Table of Contents

S1. Syntheses……S2

S2. MCXRD data collection, data processing pipeline, crystal structure determination and structure refinement……S3

S2.1 Sample mounting for X-ray data collection……S3

S2.2 X-Ray data collection……S3

S2.3 X-Ray data processing pipeline: data reduction; data set selection and combination……S4

S2.4 Crystal structure determination and refinement details……S5

S3. Multi-crystal data processing summaries……S12

S3.1 Data collection and data processing summaries……S12

S3.2 Crystallographic statistics for final merged data sets (overall and by resolution shell)……S16

S3.3 Crystallographic statistics for data sets from individual crystals……S20

S4. *RADDOSE-3D* X-ray dose calculations for MOF-919(Sc/Cu)……S25

S4.1 X-Ray fluorescence dose measurements and calculations for MOF-919(Sc/Cu)……S25

S4.2 X-Ray dose calculations for data collections used for crystal structure determination of MOF-919(Sc/Cu) and beamline VMXm setup……S25

S5. Comparison of stationary-MCXRD (SX), rotation-MCXRD and SCXRD crystal structures of MOF-919(Sc/Cu)……S26

S6. References……S29

S1. Syntheses

The MOFs UiO-66,[S1,S2] MET-2,[S3] and MOF-919(Sc/Cu)[S4] were synthesised according to reported procedures, yielding crystals of comparable size to those originally reported. UiO-66-MoO_4 was prepared by post-synthetic modification of UiO-66 with retention of crystal size. PCN-260(Sc) and MIL-88B(Cr)-1,4-NDC have not previously been reported.

PCN-260(Sc)

$Sc(NO_3)_3 \cdot 4H_2O$ (30 mg, 0.1 mmol) and 1,3,5-tris(4-carboxyphenyl)benzene (43.8 mg, 0.1 mmol) were added to a 25 mL vial. *N*,*N*-dimethylacetamide (2.8 mL, 30 mmol, 300 equivalents) and HCl (61 μL, 2 mmol) were added to the vial. The vial was sonicated for 10 mins to dissolve all the material before being placed in an oven at 120 °C. After 24 h, the vial was removed and allowed to cool to room temperature. Crystals formed as colourless rectangular prisms (Figure 2a) and were used for MCXRD studies in their as-synthesised form.

$[Cr_3O(O_2CCH_3)_6(H_2O)_3]NO_3 \cdot 6H_2O$

$Cr(NO_3)_3 \cdot 9H_2O$ (12 g, 0.03 mol) was dissolved in 10 mL hot water (50 °C). $CH_3COONa \cdot 3H_2O$ (8.16 g, 0.06 mol) was dissolved separately in 10 mL hot water (50°C). The solutions were mixed and stirred at 50 °C for 10 mins before leaving to crystallise at room temperature. After 1-2 weeks, dark green crystals of the $[Cr_3O(O_2CCH_3)_6(H_2O)_3]NO_3 \cdot 6H_2O$ product were formed. These were collected by filtration for use in synthesis of MIL-88B(Cr)-1,4-NDC.

MIL-88B(Cr)-1,4-NDC

1,4-Naphthalenedicarboxylic acid (216 mg, 1 mmol) and $[Cr_3O(O_2CCH_3)_6(H_2O)_3]NO_3 \cdot 6H_2O$ (250 mg, 0.33 mmol) were loaded into a 50 mL Pyrex jar. Water (4 mL), pyridine (6 mL) and glacial acetic acid (0.572 mL, 10 mmol) were added. The mixture was sonicated until the solids were fully dissolved, then transferred to a 23 mL capacity Teflon-lined stainless-steel autoclave and sealed tightly before placing it in an oven at 220 °C. After heating for 24 h, the autoclave was removed and allowed to cool to room temperature. Crystals formed as dark green bicapped hexagonal prisms (Figure 2a) and were collected by centrifugation and washed 3 times with DMF (20 mL), separating by centrifugation each time. These crystals were used for MCXRD studies in their as-synthesised form, although structure determination suggests some solvent loss has occurred.

UiO-66

Zirconium chloride (303 mg, 1.30 mmol), terephthalic acid (224 mg, 1.33 mmol) and benzoic acid (4.7 g, 38.5 mmol) were dissolved in DMF (10 mL) which had been dried using a Grubbs solvent system.[S5] The solution was placed in a conical flask which had been pre-treated by soaking overnight with a saturated aqueous solution of KOH. A loose cap was placed on the flask to allow evaporation of volatile by-products and the solution was heated without stirring at 130 °C. Colourless microscopic crystals began to appear after 24 h and heating was continued for a further 24 h. After cooling to room temperature, the remaining solvent was decanted and the crystals washed with 5 x 10 mL portions of fresh DMF.

UiO-66-MoO_4

Crystals of UiO-66, synthesised as described above and stored in fresh DMF, were used for post-synthetic modification to introduce molybdate sites. The DMF solvent was decanted and replaced with a solution of sodium molybdate in water (10 mL, 20 mM) and heated to 60 °C for 24 h. After cooling to room temperature, the solvent was replaced with water and kept in solution until analysis by MCXRD. The colourless octahedral crystals are shown in Figure 2a.

MET-2

$Mn(NO_3)_2 \cdot 4H_2O$ (0.251 g, 1 mmol) was dissolved in DMF (10 ml) in a Pyrex beaker, then 1H-1,2,3-triazole (0.173 g, 2.5 mmol) was decanted into this solution. The resulting mixture was decanted into a glass vial.

The vial was capped and placed into a preheated oven at 120 °C for 24 h, then cooled to 25 °C at a rate of 0.1 °C min^{-1}. MET-2 formed as a colourless micro-crystalline powder, which was then washed 3 times with DMF. These octahedral microcrystals (Figure 2a) were then used in this as-synthesised form for MCXRD measurements. The synthetic procedure is slightly modified from that originally reported[S3] by use of DMF instead of *N*,*N*-diethylformamide (DEF) as solvent.

MOF-919(Sc/Cu)

Synthesis was carried out by following the procedure reported by Liu *et al.,*[S4] yielding pale blue-green octahedral crystals of MOF-919(Sc/Cu) (Figure 2a). Crystals were solvent-exchanged multiple times with fresh DMF and removed from solvent in this as-synthesised form for use in rotation-MCXRD and stationary-MCXRD (SX) studies.

S2. MCXRD data collection, data processing pipeline, crystal structure determination and structure refinement

S2.1 Sample mounting for X-ray data collection

Crystals of MOFs used in diffraction studies were imaged using a field-emission scanning electron microscope (JEOL JSM-IT100 InTouchScope).

For data collection at beamlines I04 and I24, crystal slurries were pipetted onto a Mylar film[S6] and covered with a second such film to retain crystal solvation. For data collection at beamline VMXm crystal slurries were pipetted onto copper-mesh QUANTIFOIL® holey carbon supported cryo-electron microscopy (cryoEM) grids.[S7] Excess solvent was permitted to evaporate before mounting the grids into VMXm sample mounts for room temperature (293 K) data collection. For crystals where such solvent loss led to substantial reduction in X-ray data quality crystals were prepared using a Leica EM GP2 automated plunge freezing device. Crystals were pipetted with excess solvent onto cryoEM grids then blotted on the reverse side with blotting paper before vitrification in liquid ethane.[S8] Loaded grids were then stored in cryoEM grid boxes and pucks under liquid nitrogen prior to mounting on the beamline for low temperature (120(10) K) X-ray data collection. The grids were inspected and the crystal size was assessed using a laboratory-based SEM instrument fitted with a cryo-stage.

S2.2 X-Ray data collection

X-Ray data were collected for PCN-260(Sc) and MIL-88(Cr)-NDC at 293 K by rotation-MCXRD at beamline I24 using an Eiger 9M CdTe detector and synchrotron X-ray radiation (λ = 0.5390 Å; beamsize 8 μm x 8 μm). Individual crystals of the MOFs were located in the Mylar sheet and data collected by rotation of the diffractometer φ-axis as a series of 0.1° wide frames with exposure times of 0.05-0.1 s/frame (Tables S2-S7). Data were collected for 54 crystals of PCN-260(Sc) and 19 crystals of MIL-88(Cr)-NDC. Data were collected in analogous manner for UiO-66 (50 crystals) and UiO-66-MoO_4 (76 crystals) at 293 K at beamline I04, Diamond Light Source, using a Dectris Pilatus2 6MF detector and synchrotron X-ray radiation (λ = 0.7749 Å; beamsize 5 μm x 10 μm (v x h)) with exposure times of 0.3 s/frame for UiO-66 and 0.15 s/frame for UiO-66-MoO_4 for 0.2° wide frames. Data were collected in analogous manner (rotation-MCXRD) for MET-2 (71 crystals) at 293 K at beamline VMXm, Diamond Light Source, using an Eiger 9M CdTe detector and synchrotron X-ray radiation (λ = 0.4979 Å; beamsize 1.2 μm x 3.8 μm (v x h)) with exposure times of 0.2-0.5 s/frame for 0.1° wide frames. Data were collected by both rotation-MCXRD (117 crystals, 0.1° frames) and stationary-MCXRD (SX) methods for MOF-919(Sc/Cu) at 120(10) K at beamline VMXm, using an Eiger 9M CdTe detector and synchrotron X-ray radiation (λ = 0.4979 Å; beamsizes 1.3-2.8 μm x 2.9-3.7 μm (v x h)) with exposure times of 1.5-3 s/frame

for rotation-MCXRD and 2-4 s/frame for stationary-MCXRD (SX). SX data were measured for 3023 grid locations (grid size 5 x 5 μm), not all of which may contain crystals.

S2.3 X-Ray data processing pipeline: data reduction; data set selection and combination

The data processing pipeline developed for the rotation-MCXRD studies (summarised in Figure 1) has been adapted from macromolecular crystallography through choice of software parameters; automation has been augmented by locally written scripts. Specific command instructions used to optimise software performance for chemical crystallography are presented for each study in Section 3. Data reduction for rotation-MCXRD data was conducted in an automated manner using the *xia2* programs *xia2.dials*[S9] and *xia2.multiplex*,[S10] which direct data reduction via the *dials* suite of programs.[S11,S12] *xia2.dials* operates on data sets from individual crystals, whereas *xia2.multiplex* is a multi-crystal and multi-analysis pipeline. Initial spot finding, indexing and integration of reflections for each crystal was undertaken with *xia2.dials*. Spot-finding parameters were matched to the size of the recorded spots and for data collected at beamline VMXm. A reflection mask to obscure Debye-Scherrer rings that arise from the copper components of the cryoEM grids was applied. The ensemble of integrated partial data sets was analysed with *xia2.multiplex*, initially to perform clustering of data with similar unit cell parameters, undertake Bravais lattice and Laue group determination, and determine the (best) average unit cell parameters for use in subsequent analyses. If multiple distinct clusters of data sets were identified, the most populated cluster was taken forward, but other clusters can be examined subsequently. In all examples herein, a single prominent cluster was identified and other data sets were removed as non-conforming outliers. Multi-crystal scaling, using *dials.scale*[S11,S12] within *xia2.multiplex*, was then applied to merge all data sets in conjunction with application of an empirical correction of diffraction intensities for X-ray absorption and other systematic errors. This was followed by space group determination (or direct input) for the merged data set.

Multi-crystal filtering methods were then applied to remove sequentially either the least conformative data sets, based on the $\Delta CC_{1/2}$ method,[S13,S14] or the lowest quality data set, based on R_{pim} values[S15,S16] for the individual data sets, as determined by *xia2.dials.* After each application of the assessment criterion and removal of the selected data set, the remaining data sets were combined to give a merged data set. Continuing this process sequentially yielded $N - 1$ possible merged data sets (where N = total no. of data sets collected, *i.e.* the maximum number of data sets that could be merged). The best choice of these $N - 1$ merged data sets for use in crystal structure determination was selected as that having the lowest R_{pim} value for overall data completeness > 95%. Both filtering methods ($\Delta CC_{1/2}$ or R_{pim}) were trialled for each study and the method that provided a merged data set with the most favourable statistics was adopted in each case. These approaches are described in more detail in the Supporting Information.

To assess whether a data resolution cutoff lower than the detector limit was needed, $CC_{1/2}$ values were determined for resolution shells. MIL-88B(Cr)-1,4-NDC, UiO-66, UiO-66-MoO_4 and MET-2 diffracted sufficiently strongly simply to allow the detector limit to be used, as $CC_{1/2}$ values exceeded 0.9 for the highest resolution shell, leading to resolutions (d_{min}) of 0.74-0.85 Å (Tables S10, S12, S14 and S16). Although PCN-260(Sc) crystals were the largest studied, diffraction quality was poorer. Thus, a data resolution cutoff was applied using $CC_{1/2} \geq 0.2$ for the highest resolution shell ($CC_{1/2}$ = 0.22 for $0.92 \geq d \geq 0.90$ Å), leading to d_{min} = 0.9 Å. For the rotation MCXRD data of MOF-919(Sc/Cu) the resolution was again set at a $CC_{1/2}$ value of 0.2 in the highest resolution shell ($1.17 \geq d \geq 1.15$ Å; d_{min} = 1.15 Å). The same resolution (d_{min} = 1.15 Å; $CC_{1/2}$ = 0.22 for $1.17 \geq d \geq 1.15$ Å) was then used for the SX (stationary-MCXRD) data to ensure fair model-to-model comparisons for the MOF-919(Sc/Cu) crystal structure.

Data reduction for stationary-MCXRD (SX) data for MOF-919(Sc/Cu) cannot be conducted using the same data processing pipeline used for rotation-MCXRD data as the single-image data sets for each crystal irradiated present a greater challenge for indexing, integration and merging. Data processing was conducted using established protocols using the *xia2.ssx* program,[S17] aided by the space group and approximate unit cell dimensions from the reported PXRD study.[S4] Data from 1763 grid locations were successfully indexed and were merged to give the final overall data set.

Full summaries of measurement and data quality statistics for all indexed data, and specification of data selected for use in final merged data sets is provided in Tables S2-S27.

S2.4 Crystal structure determination and refinement details

Using the best merged data set, as determined within the data processing pipeline, crystal structures were solved either by direct methods using *SHELXS*[S18] or by dual-space methods using SHELXT,[S19] except for MOF-919(Sc/Cu), where the reported PXRD structure[S4] was used as an initial model. Least-squares refinement of the models against all F^2 values were conducted using *SHELXL*[S20] implemented by *OLEX2*.[S21] Anisotropic displacement parameters were used in refinement of most non-hydrogen atoms, whereas hydrogen atoms were, in all structures except MET-2, added in calculated positions and refined using a riding model with fixed isotropic displacement parameters. For MET-2, hydrogen atoms could be located directly from the electron density difference map and their positional parameters refined freely. Restraints and occasionally constraints were needed in many of the refinements and a solvent mask was applied in the final stages of refinement, for all structures except UiO-66 and UiO-66-MoO_4, by using the *BYPASS*[S22] solvent masking function in *OLEX2*. Full details of refinement for all crystal structures are provided in this Section and crystal data are provided in Table S1.

In Section S3, data collection and data processing are documented in greater detail in tabulated form and full details can be found in the deposited CIFs (CSD deposition numbers 2487825, PCN-260(Sc); 2487826, MIL-88B(Cr)-1,4-NDC; 2487827, UiO-66; 2487828, UiO-66-MoO_4; 2487829, MET-2; 2487830, MOF-919(Sc/Cu) from rotation-MCXRD and 2487831, MOF-919(Sc/Cu) from stationary-MCXRD (SX))..

S2.4.1 PCN-260(Sc)

Multi-crystal X-ray diffraction (rotation-MCXRD) data were collected at 293 K at beamline I24,[S23] Diamond Light Source, using an Eiger 9M CdTe detector and X-rays of wavelength, λ = 0.5390 Å. Individual datasets were indexed and reflection intensities integrated using the *xia2.dials*[S9] program. The unit cell parameters were noted to resemble those of PCN-260(Fe_2Co)[S24] and reported space group $Pca2_1$ was then used to guide further data processing. Integrated reflection intensities were scaled and merged with the *dials.scale*[S11,S12] program using a physical scaling model and spherical harmonic absorption corrections. The structure was solved using direct methods using *SHELXS*[S9S18] and refined against all F^2 values using *SHELXL*,[~~S10~~S20] implemented via the *OLEX2*[S21] program *(version1.5)*. Non-hydrogen atoms were refined with anisotropic displacement parameters. Hydrogen atoms were added at calculated positions and refined by using a riding model with fixed isotropic displacement parameters. Hydrogen atoms on the terminal water and hydroxide ligands were modelled with fixed occupancies of ⅚ to account for the statistical disorder between these ligand sites. The structure was refined as a racemic twin (Flack x = 0.44(5)). A solvent mask was applied by using the *BYPASS*[S22] function implemented in *OLEX2*. Crystal data can be found in Table S1 and full details are deposited in the Crystallographic Information File (CIF). The crystal structure is shown in Figures 2c and 3a.

S2.4.2 MIL-88B(Cr)-1,4-NDC

Multi-crystal X-ray diffraction (rotation-MCXRD) data were collected at 293 K at beamline I24,[S23] Diamond Light Source, using an Eiger 9M CdTe detector and X-rays of wavelength, λ = 0.5390 Å. Individual datasets

were indexed and reflection intensities integrated using the *xia2.dials*[S9] program and the integrated reflections were passed to the *xia2.multiplex*[S10] program which employs *dials.scale*[S11,S12] to merge and rescale the data using a physical scaling model and spherical harmonic absorption corrections. The structure was solved with dual-space methods using *SHELXT*[S19] and refined against all F^2 values using *SHELXL*[S18] implemented by the *OLEX2* program (*version1.5*). Non-hydrogen atoms were refined with anisotropic displacement parameters. The naphthalene ring (atoms C2−C11) was added as a pre-made fragment using the *FRAGMENT-DB*[S25] plugin as implemented by the *OLEX2-v1.5* program and refined as a rigid body with C10 as the pivot atom. Hydrogen atoms were added at calculated positions and refined by using a riding model with fixed isotropic displacement parameters. Hydrogen atoms on the terminal water and hydroxide ligands were modelled with fixed occupancies of ⅚ to account for the statistical disorder between these ligand sites. The naphthalene ring is disordered over 4 orientations related by a 4-fold axis. A solvent mask was applied by using the *BYPASS*[S22] program implemented in *OLEX2*. Crystal data can be found in Table S1 and full details are deposited in the CIF. The crystal structure is shown in Figures 2c, 3b and 3c and corresponds to an open state of the MOF, although not at its maximum opening, and has similar unit cell dimensions to the as-synthesised MOF studied by PXRD. The MOF exhibits a continuous breathing behaviour as a function of pore-solvent content, which has been established by PXRD experiments described in further detail elsewhere[S26] and summarised in (Figure S1).

(a)

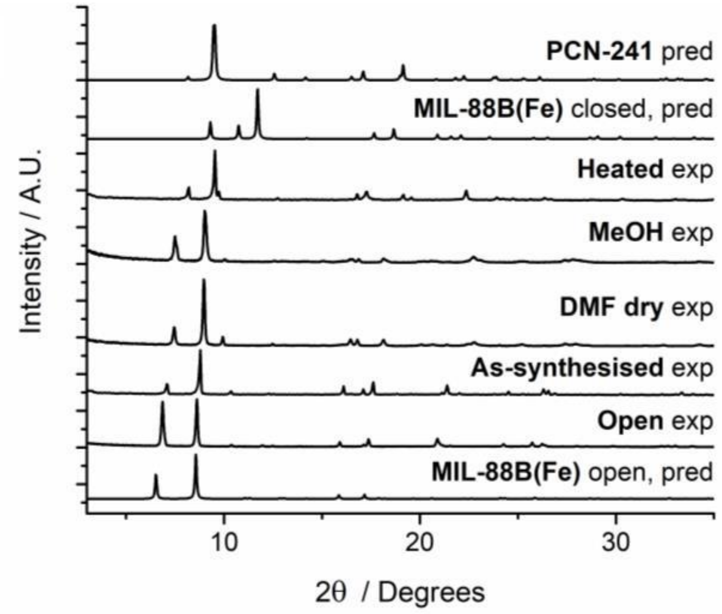

(b)

Material	Label (in Fig. S1a)	Unit cell parameters		
		a **(Å)**	***c*** **(Å)**	***V*** **(Å³)**
**MIL-88B(Cr)-1,4-NDC** open-pore	**Open** exp	14.880	16.956	3251.3
**MIL-88B(Cr)-1,4-NDC** as-synthesised	**As-synthesised** exp	14.398	17.103	3070.2
**MIL-88B(Cr)-1,4-NDC** heated	**Heated** exp	12.467	18.157	2443.9
**MIL-88B(Cr)-1,4-NDC** with MeOH as pore solvent	**MeOH** exp	13.583	17.635	2817.5
**MIL-88B(Cr)-1,4-NDC** with dry DMF as pore solvent	**DMF dry** exp	13.841	17.317	2872.9
**MIL-88B(Fe)-1,4-BDC** open-pore[S27]	**MIL-88B(Fe)** open, pred	15.626	15.960	3375.0
**MIL-88B(Fe)-1,4-BDC** closed-pore[S27]	**MIL-88B(Fe)** closed, pred	9.600	19.100	1524.4
**PCN-241** (*i.e.* MIL-88(Fe_2Co)-1,4-NDC)[S24]	**PCN-241** pred	12.943	18.533	2505.0
**MIL-88B(Cr)-1,4-NDC** MCXRD study (this work)	n.a.	14.117	17.201	2968.8

Figure S1. (a) Experimental PXRD patterns for MIL-88(Cr)-1,4-NDC in different states of solvation and calculated patterns for comparable MIL-88B(Fe)[S26] (*i.e.* MIL-88(Fe)-1,4-BDC) and PCN-241[S24] (*i.e.* MIL-88(Fe_2Co)-1,4-NDC) MOFs. (b) Unit cell parameters determined from Pawley fitting[S28] of PXRD patterns[S26] for MIL-88(Cr)-1,4-NDC in different states of solvation and noted for comparison for MIL-88B(Fe) (*i.e.* MIL-88(Fe)-1,4-BDC) and PCN-241 (*i.e.* MIL-88(Fe_2Co)-1,4-NDC) and the present MCXRD study of MIL-88(Cr)-1,4-NDC.

S2.4.3 UiO-66

Multi-crystal X-ray diffraction (rotation-MCXRD) data were collected at 293 K at beamline I04,[S29] Diamond Light Source, using a Dectris Pilatus2 6MF detector and X-rays of wavelength, λ = 0.7749 Å. Individual datasets were indexed and reflection intensities integrated using the *xia2.dials*[S9] program and the integrated reflections were passed to the *xia2.multiplex*[S10] program which utilises *dials.scale*[S11,S12] to merge and rescale the data using a physical scaling model and spherical harmonic absorption corrections. The known space group and unit cell parameters were used to guide processing. The structure was solved with dual-space methods using *SHELXT*[S19] and refined against all F^2 values using *SHELXL*[S20] implemented by the *OLEX2*[S21] program (*version1.5*). Non-hydrogen atoms were refined with anisotropic displacement parameters. The μ_3-O and μ_3-OH oxygen atoms were assigned fixed occupancies of 0.5 to account for the site disorder. Hydrogen atoms were added at calculated positions and refined using a riding model with fixed isotropic displacement parameters. Crystal data can be found in Table S1 and full details are deposited in the CIF. The crystal structure is shown in Figures 2c and S2.

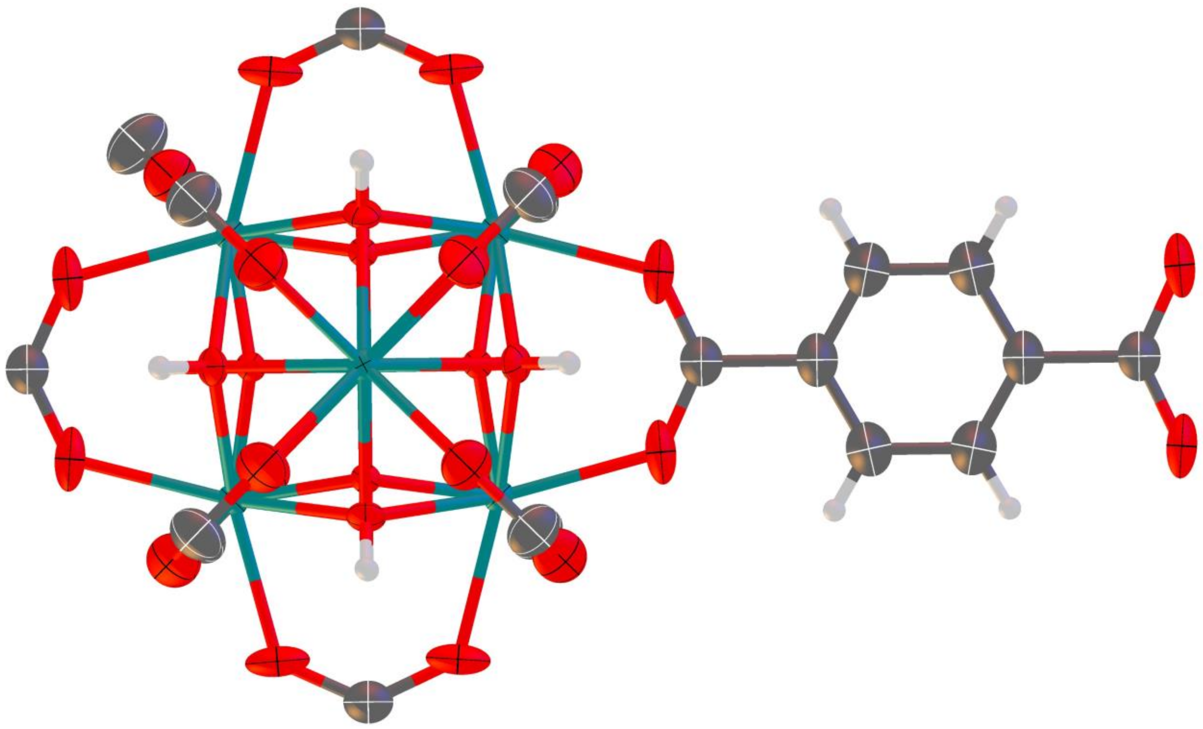

Figure S2. Crystal structure of UiO-66 showing $Zr_6(\mu_3\text{-}O)_4(\mu_3\text{-}OH)_4(O_2CR)_{12}$ secondary building unit and 1,4-BDC linker ligand. Zr atoms, green; C atoms, grey, O atoms, red shown with atoms represented as 50% displacement ellipsoids. H atoms represented as pale grey spheres of arbitrary radius.

S2.4.4 UiO-66-MoO_4

A preliminary analysis of the MCXRD data and structure determination was presented at the MOF2016 conference.[S30] These data have been reanalysed using the data processing pipeline presented in this work. Multi-crystal X-ray diffraction (rotation-MCXRD) data were collected at 293 K at beamline I04,[S29] Diamond Light Source, using a Dectris Pilatus2 6MF detector and X-rays of wavelength, λ = 0.7749 Å.

Individual datasets were indexed and reflection intensities integrated using the *xia2.dials*[S9] program and the integrated reflections were passed to the *xia2.multiplex*[S10] program which utilises *dials.scale*[S11,S12] to merge and rescale the data using a physical scaling model and spherical harmonic absorption corrections. The known space group and unit cell parameters were used to guide processing. The structure was solved with direct methods using *SHELXS*[S18] and refined against all F^2 values using *SHELXL*[S20] implemented by the *OLEX2*[S21] program (*version1.5*). The crystal structure is shown in Figures 4a and S3. Non-hydrogen framework atoms (Zr1, C1−C3, O1−O3) were refined with anisotropic displacement parameters. Atoms O1 and O2 have occupancies set at 0.5 to represent the 50:50 site disorder of the oxide and hydroxide ligands (Figure S3). The molybdate anion (atoms Mo1, O4−O6) was added to the refinement model with a geometry based upon a similarly coordinated molybdate ion.[S31] The initial position of Mo1 was determined from the difference electron density peak assignable to the Mo atom (Figure 4b) and the initial orientation of the molybdate ion was based upon the coordinated molybdate in the previously reported NU-1000 MOF.[S31] The geometry of the molybdate fragment was refined with geometric restraints and oxygen atoms were assigned fixed isotropic displacement parameters constrained to 1.2 times that of the isotropic displacement parameter refined for the Mo atom. A single (SHELXL) free variable was used to refine the occupancy of the disordered BDC and the molybdate anion such that the total site occupancy was equal to unity resulting in a BDC:molybdate ratio of 0.810(11):0.190(11). It is evident from the structure missing terephthalate ligands will not be replaced by two molybdate ions as there is not space to accommodate two ions, as evident from the disorder model (Figure S3c), nor would this retain the overall charge balance. Thus we anticipate that each missing terephthalate ligand is replaced by a coordinated molybdate at the edge site of one Zr_6 cluster and two water molecules at the edge site of the other Zr_6 cluster previously connected by a terephthalate ligand. The water molecules were not located in the electron density map and are not included in the refined structure model. Hydrogen atoms were added at calculated positions and refined using a riding model with fixed isotropic displacement parameters. Crystal data can be found in Table S1 and full details are deposited in the CIF.

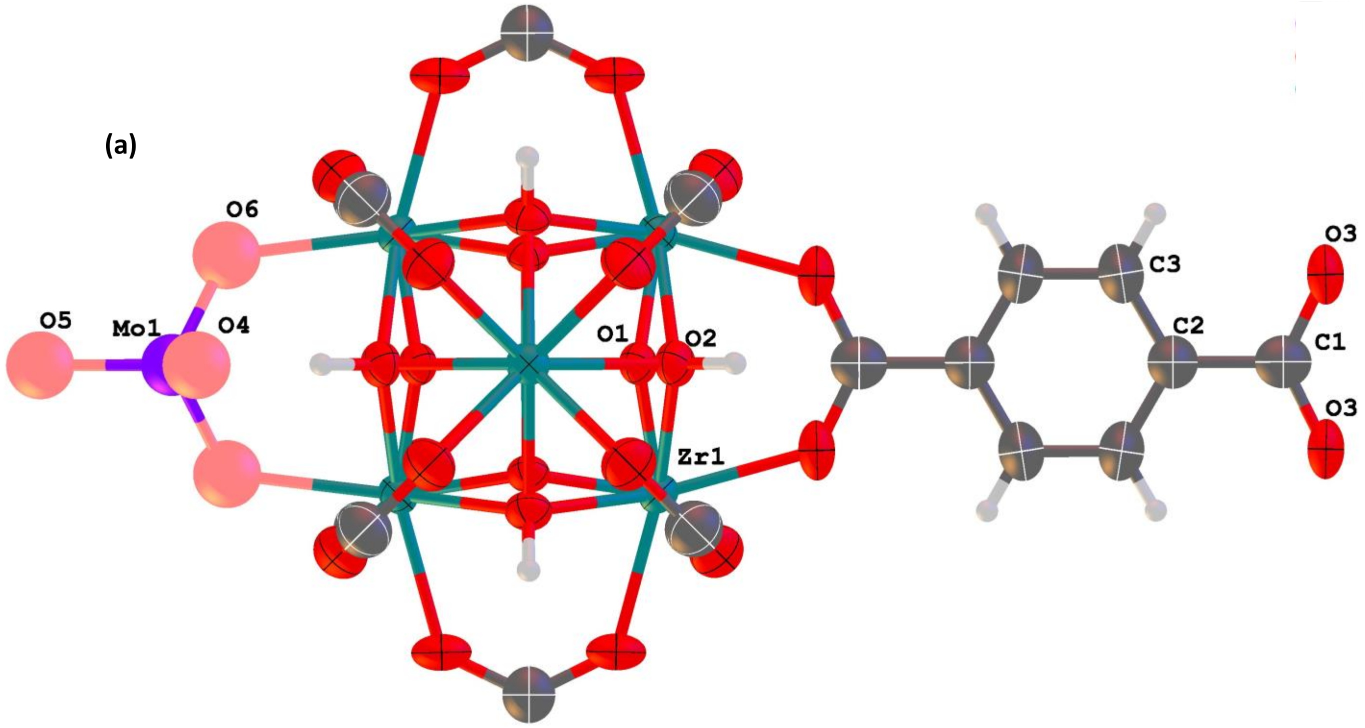

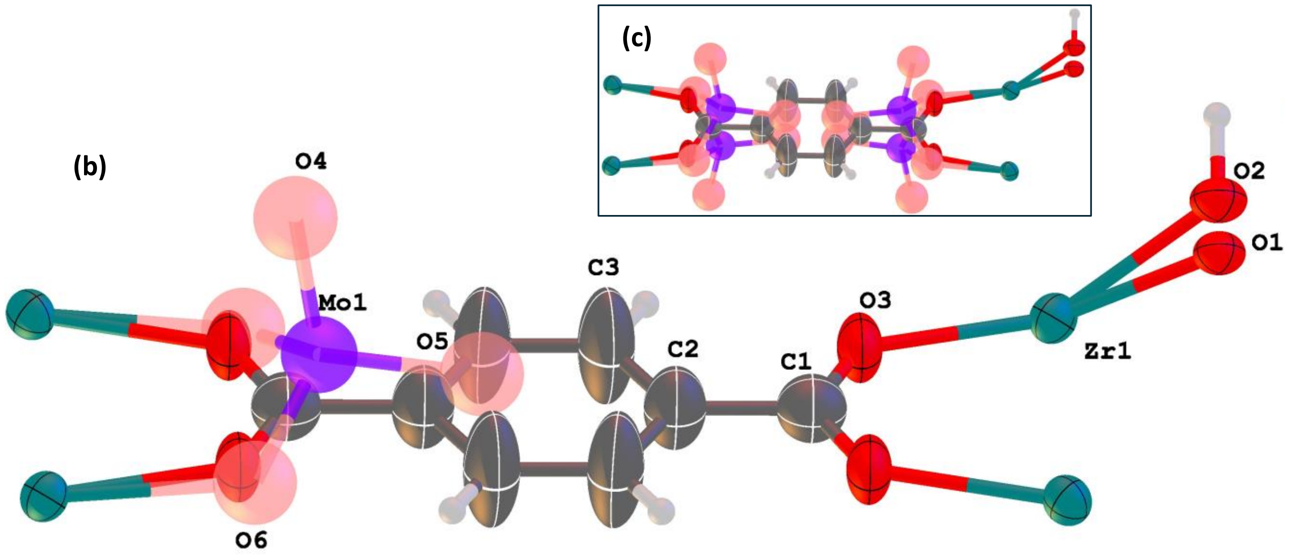

Figure S3. Crystal structure of UiO-66-MoO$_4$ shown with non-hydrogen atoms represented as 50% displacement ellipsoids (molybdate atoms with isotropic displacement parameters). (a) Representation of $Zr_6(\mu_3\text{-}O)_4(\mu_3\text{-}OH)_4$ SBU and a single coordinated terephthalate and molybdate shown, for clarity. Positional disorder of terephthalate ligand and (superposed semi-transparent) molybdate group is shown in (b) with a single MoO_4^{2-} unit represented and in (c) [inset] with the four symmetry-related MoO_4^{2-} sites shown. Zr atoms, green; C atoms, grey, O atoms, red/pink; Mo atoms purple. Positional disorder of oxide and hydroxide moieties (atoms labelled O1 and O2, respectively) is shown in all images.

S2.4.5 MET-2

Multi-crystal X-ray diffraction (rotation-MCXRD) data were collected at 293 K at beamline VMXm,[S32] Diamond Light Source, using an Eiger 9M CdTe detector and X-rays of wavelength, λ = 0.4979 Å. Individual datasets were indexed and reflection intensities integrated using the *xia2.dials*[S9] program and the integrated reflections were passed to the *xia2.multiplex*[S10] program which utilises *dials.scale*[S11,S12]to merge and rescale the data using a physical scaling model and spherical harmonic absorption corrections. The known space group and unit cell parameters were used to guide processing. The structure was solved with dual-space methods using *SHELXT*[S19] and refined against all F^2 values using *SHELXL*[S20] implemented via the *OLEX2*[S21] program (*version1.5)*. Non-hydrogen atoms were refined with anisotropic displacement parameters. Hydrogen atom H1 was located from a difference electron density map (Figure 4d) and refined with an isotropic displacement parameter. A solvent mask was applied by using the *BYPASS*[S22] program as implemented in *OLEX2-v1.5*. Crystal data can be found in Table S1 and full details are deposited in the CIF. The crystal structure is shown in Figures 2c and 4c.

S2.4.6 MOF-919(Sc/Cu)

Multi-crystal X-ray diffraction data (both rotation-MCXRD and stationary-MCXRD (SX)) were collected at 120(10) K at beamline VMXm,[S32] Diamond Light Source, using an Eiger 9M CdTe detector and X-rays of wavelength, λ = 0.4979 Å. Individual rotation-MCXRD data sets were indexed and reflection intensities integrated using the *xia2.dials*[S9] program and the integrated reflections were passed to the *xia2.multiplex*[S10] program which utilises *dials.scale*[S11,S12] to merge and rescale the data using a physical scaling model and spherical harmonic absorption corrections. Stationary-crystal (SX, *i.e.* single-image) data were processed using the *xia2.ssx*[S17] program which utilises *dials.scale* to merge and rescale the data using a KB scaling model. The known space group and unit cell parameters were used to guide processing. The atom coordinates from the previously reported structure model based upon Rietveld refinement of PXRD data[S4,S33] were used as a starting point for crystal structure refinement and completion of the crystal structure was achieved by atom assignment from difference electron density maps. All least-

squares refinement of the structure model was carried out against all F^2 values using *SHELXL*[S20] implemented by the *OLEX2*[S21] program (*version1.5)*. The structure model was initially refined against the merged rotation-MCXRD data set and resulted in a much improved structure model (see Section S5). The improved model resulting from this refinement was then used as a starting model for refinement against the merged stationary-MCXRD (SX) data set. For refinement of models against both data sets, non-hydrogen atoms were refined with anisotropic displacement parameters. Hydrogen atoms were added at calculated positions and refined by using a riding model with fixed isotropic displacement parameters. A solvent mask was applied by using the *BYPASS*[S22] program as implemented in *OLEX2-v1.5*. Crystal data for both structure determinations can be found in Table S1 and full details are deposited in the CIFs. The crystal structures are shown in Figures 2c and 5a and are discussed in comparison with the recently reported SCXRD structure in Section S5.

Table S1. Data collection, structure solution and refinement parameters for X-ray structure determinations of PCN-260(Sc), MIL-88B(Cr)-1,4-NDC, UiO-66, UiO-66-MoO$_4$, MET-2 and MOF-919(Sc/Cu).

	**PCN-260(Sc)**	**MIL-88B(Cr)-1,4-NDC**	**UiO-66**	**UiO-66-MoO$_4$**	**MET-2**	**MOF-919(Sc/Cu)**	**MOF-919(Sc/Cu)**
**Chemical formula**	$C_{54}H_{35}O_{16}Sc_3$	$C_{36}H_{38}O_{16}Cr_3$	$C_{48}H_{28}O_{32}Zr_6$	$C_{38.88}H_{23.44}O_{32}Mo_{1.14}Zr_6$	$C_4H_4N_6Mn$	$C_{24}H_{27}N_{12}O_{24}Cu_6Sc_3$	$C_{24}H_{27}N_{12}O_{24}Cu_6Sc_3$
**Data collection**	Rotation-MCXRD	Rotation-MCXRD	Rotation-MCXRD	Rotation-MCXRD	Rotation-MCXRD	Rotation-MCXRD	Stationary-MCXRD (SX)
**Filtering method**	R_{pim}	$\Delta CC_{1/2}$	R_{pim}	R_{pim}	$\Delta CC_{1/2}$	$\Delta CC_{1/2}$	None
**No. datasets merged**	4	6	10	20	25	39	1763
**Crystal habit**	Rectangular prism	Bicapped hexagonal prism	Octahedron	Octahedron	Octahedron	Octahedron	Octahedron
**Crystal Colour**	Colourless	Dark green	Colourless	Colourless	Colourless	Pale blue-green	Pale blue-green
**Ave. crystal size (μm)**	20 x 8 x 3	15 x 7 x 3	5 x 5 x 5	5 x 5 x 5	1.1 x 0.7 x 0.7	4 x 4 x 4	4 x 4 x 4
**Crystal System**	Orthorhombic	Hexagonal	Cubic	Cubic	Cubic	Cubic	Cubic
**Space Group, *Z***	$Pca2_1$, 8	$P6_3/mmc$, 2	*Fm*-3*m*, 4	*Fm*-3*m*, 4	*Fd*-3*m*, 24	*Fd*-3*m*, 272	*Fd*-3*m*, 272
***a* (Å)**	36.2491(4)	14.1170(4)	20.76730(10)	20.7409(9)	18.1451(13)	114.356(3)	114.4(3)[a]
***b* (Å)**	18.7235(1)	14.1170(4)	20.76730(10)	20.7409(9)	18.1451(13)	114.356(3)	114.4(3)[a]
***c* (Å)**	49.2273(1)	17.2012(5)	20.76730(10)	20.7409(9)	18.1451(13)	114.356(3)	114.4(3)[a]
**α (°)**	90	90	90	90	90	90	90
**β (°)**	90	90	90	90	90	90	90
**γ (°)**	90	120	90	90	90	90	90
***V* (Å^3)**	33410.8(6)	2968.75(15)	8956.54(8)	8922.4(7)	5974.2(7)	1495459(68)	1498313(6804)[a]
**Solvent mask**	Yes	Yes	No	No	Yes	Yes	Yes
**X-ray wavelength, λ (Å)**	0.5390	0.5390	0.7749	0.7749	0.4979	0.4979	0.4979
**Beamline (DLS)**	I24	I24	I04	I04	VMXm	VMXm	VMXm
**Resolution, d_{min} (Å)**	0.90	0.74	0.80	0.80	0.85	1.15	1.15
**Temperature (K)**	293	293	293	293	293	120(10)	120(10)
***F*(000)**	4400	908	3248	3176	2280	186048	186048
**Reflns collected**	132810	19681	14049	24513	5433	318212	893285
**Independent reflns**	43557	1342	515	515	266	22150	22244
**R_{meas}**	0.203	0.066	0.055	0.064	0.139	0.358	-
**R_{pim} (R_{split})**	0.083	0.016	0.011	0.010	0.027	0.090	0.067 (0.092)
**$CC_{1/2}$**	0.989	0.999	0.997	0.999	0.992	0.979	0.994
**Flack parameter**	0.44(5)	-	-	-	-	-	-
**Mean *I*/σ(*I*)[b]**	6.0	29.6	43.5	56.8	19.8	5.9	10.8
**Completeness**	0.986	0.992	1.000	1.000	0.953	0.969	0.971
**Reflns used in refinement, *n***	43557	1342	515	515	266	22150	22244
**L.S. parameters, *p***	1081	92	30	40	23	875	875
**No. of restraints, *r***	158	66	0	9	0	1102	1102
**$R1(F)^c$ $I > 2\sigma(I)$**	0.0603	0.0808	0.0588	0.0436	0.0306	0.1037	0.1231
**$wR2(F^2)^c$, all data**	0.1589	0.2681	0.2273	0.1787	0.0869	0.3147	0.3708
**$S(F^2)^c$, all data**	0.851	1.075	1.325	1.342	1.060	1.005	1.243

a Median unit cell parameter, *a*, with its sample standard deviation listed as its associated error value determined by *xia2.ssx*.[S17] Unit cell volume, *V*, is calculated from median value of *a*, with its associated error calculated by *OLEX2* from propagation of error in *a*.

b Mean I/σ(I) values, calculated here by *OLEX2*, differ slightly from those calculated by *xia2* and reported in Tables S8, S10, S12, S14, S16, S18, S20.

c

$$R1(F) = \sum |F_0| - |F_c| / \sum |F_0|; \quad wR2(F^2) = \sqrt{\sum w(F_0^2 - F_c^2)^2 / \sum wF_0^4}; \quad S(F^2) = \sqrt{\sum w(F_0^2 - F_c^2)^2 / (n + r - p)}$$

S3. Multi-crystal data processing summaries

S3.1 Data collection and data processing summaries

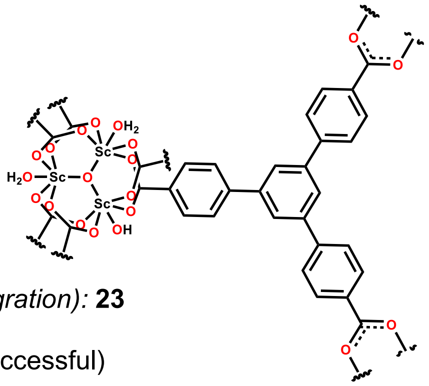

S3.1.1 PCN-260(Sc)

Number of crystals for which data collected: **54**
Number of data sets passing the xia2.dials processing stage (indexing, integration): **23**
Maximum number of mergeable datasets before filtering: **18**
Filtering method: $\boldsymbol{R}_{\mathbf{pim}}$ (undertaken manually as *xia2.multiplex* filtering not successful)
Number of omitted datasets using R_{pim} filtering: **14**
Number of data sets combined in final merged data set: **4**

***xia2.dials* commands used:** d_max=120, small_molecule=True, trust_beam_centre=True, failover=True, keep_all_reflections=False, integrate.min_spots.overall=10, integrate.min_spots.per_degree=0, space_group=Pca21, unit_cell=36,18.7,49,90,90,90

***dials.scale* commands used:** unit_cell=36,18.7,49,90,90,90, space_group=Pca21, d_min=0.90

Table S2. Experimental details for individual rotation-MCXRD data collections for **PCN-260(Sc)**.

Crystal number	Total crystal rotation (°)	Rotation per diffraction image (°)	Exposure per image (s)	Number of images merged (omitted)	Images used
Crystal 1	50	0.1	0.05	500 (0)	1-500
Crystal 2	30	0.1	0.05	300 (0)	1-300
Crystal 3	30	0.1	0.05	300 (0)	1-300
Crystal 4	60	0.1	0.05	600 (0)	1-600

S3.1.2 MIL-88(Cr)-1,4-NDC

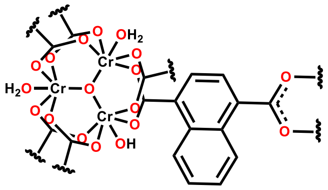

Number of crystals for which data collected: **19**
Number of datasets passing the xia2.dials processing stage (indexing, integration): **10**
Maximum number of mergeable datasets before filtering: **7**
Filtering method: $\boldsymbol{\Delta CC_{1/2}}$
Number of datasets removed using $\Delta CC_{1/2}$ filtering: **1**
Number of data sets combined in final merged data set: **6**

***xia2.dials* commands used:** small_molecule=true, trust_beam_centre=True, failover=True, keep_all_reflections=true, space_group=P63/mmc, unit_cell=14,14,17,90,90,120

***xia2.multiplex* commands used:** filtering.method=deltacchalf, deltacchalf.stdcutoff=3, resolution.d_min=0.74, symmetry.cosym.space_group=P63/mmc, symmetry.space_group=P63/mmc, unit_cell=14.1,14.1,17.2,90,90,120, unit_cell_clustering.threshold=10

Table S3. Experimental details for individual rotation-MCXRD data collections for **MIL-88(Cr)-1,4-NDC**.

Crystal number	Total crystal rotation (°)	Rotation per diffraction image (°)	Exposure per image (s)	Number of images merged (omitted)	Images used
Crystal 1	30	0.1	0.1	300 (0)	1-300
Crystal 2	30	0.1	0.1	300 (0)	1-300
Crystal 3	20	0.1	0.1	200 (0)	1-200
Crystal 4	80	0.1	0.1	800 (0)	1-800
Crystal 5	30	0.1	0.1	300 (0)	1-300
Crystal 6	40	0.1	0.1	400 (0)	1-400

S3.1.3 UiO-66

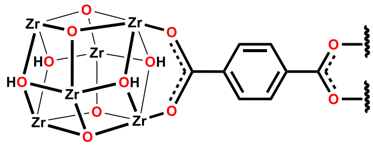

Number of crystals for which data collected: **50**
Number of datasets passing the xia2.dials processing stage (indexing, integration): **28**
Maximum number of mergeable datasets before filtering: **28**
Filtering method: $\boldsymbol{R}_{\mathbf{pim}}$
Number of omitted datasets using R_{pim} filtering: **18**

Number of data sets combined in final merged data set: **10**

***xia2.dials* commands:** d_max=120, small_molecule=true, trust_beam_centre=True, failover=True, keep_all_reflections=true, integrate.min_spots.overall=10, integrate.min_spots.per_degree=0, space_group=Fm-3m, unit_cell=20,20,20,90,90,90

***xia2.multiplex* commands:** filtering.method=deltacchalf, deltacchalf.stdcutoff=3, unit_cell_clustering.threshold=20, unit_cell=20,20,20,90,90,90, resolution.d_min=0.80, min_completeness=0.80, symmetry.cosym.space_group=Fm-3m, symmetry.space_group=Fm-3m, resolution.space_group=Fm-3m

Table S4. Experimental details for individual rotation-MCXRD data collections for **UiO-66**.

Crystal number	Total crystal rotation (°)	Rotation per diffraction image (°)	Exposure per image (s)	Number of images merged (omitted)	Images used
Crystal 1	20	0.2	0.3	100 (0)	1-100
Crystal 2	20	0.2	0.3	100 (0)	1-100
Crystal 3	20	0.2	0.3	100 (0)	1-100
Crystal 4	20	0.2	0.3	100 (0)	1-100
Crystal 5	20	0.2	0.3	100 (0)	1-100
Crystal 6	20	0.2	0.3	100 (0)	1-100
Crystal 7	20	0.2	0.3	100 (0)	1-100
Crystal 8	20	0.2	0.3	100 (0)	1-100
Crystal 9	20	0.2	0.3	100 (0)	1-100
Crystal 10	20	0.2	0.3	100 (0)	1-100

S3.1.4 UiO-66-MoO_4

Number of crystals for which data collected: **76**
Number of datasets passing the xia2.dials processing stage (indexing, integration): **69**
Maximum number of mergeable datasets before filtering: **69**
Filtering method: $\boldsymbol{R_{pim}}$
Number of omitted datasets using R_{pim} filtering: **49**
Number of data sets combined in final merged data set: **20**

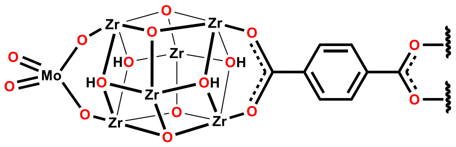

***xia2.dials* commands:** d_max=120, small_molecule=true, trust_beam_centre=True, failover=True, keep_all_reflections=true, integrate.min_spots.overall=10, integrate.min_spots.per_degree=0, space_group=Fm-3m, unit_cell=20,20,20,90,90,90

***xia2.multiplex* commands:** filtering.method=deltacchalf, deltacchalf.stdcutoff=3, unit_cell_clustering.threshold=20, unit_cell=20,20,20,90,90,90, resolution.d_min=0.80, min_completeness=0.80, symmetry.cosym.space_group=Fm-3m, symmetry.space_group=Fm-3m, resolution.space_group=Fm-3m

Table S5. Experimental details for individual rotation-MCXRD data collections for **UiO-66-MoO_4**.

Crystal number	Total crystal rotation (°)	Rotation per diffraction image (°)	Exposure per image (s)	Number of images merged (omitted)	Images used
Crystal 1	15	0.2	0.15	75 (0)	1-75
Crystal 2	20	0.2	0.15	100 (0)	1-100
Crystal 3	15	0.2	0.15	75 (0)	1-75
Crystal 4	20	0.2	0.15	100 (0)	1-100
Crystal 5	20	0.2	0.15	100 (0)	1-100
Crystal 6	15	0.2	0.15	75 (0)	1-75
Crystal 7	15	0.2	0.15	75 (0)	1-75
Crystal 8	20	0.2	0.15	100 (0)	1-100
Crystal 9	15	0.2	0.15	75 (0)	1-75
Crystal 10	20	0.2	0.15	100 (0)	1-100
Crystal 11	15	0.2	0.15	75 (0)	1-75
Crystal 12	20	0.2	0.15	100 (0)	1-100
Crystal 13	15	0.2	0.15	75 (0)	1-75
Crystal 14	15	0.2	0.15	75 (0)	1-75
Crystal 15	20	0.2	0.15	100 (0)	1-100
Crystal 16	15	0.2	0.15	75 (0)	1-75
Crystal 17	15	0.2	0.15	75 (0)	1-75
Crystal 18	15	0.2	0.15	75 (0)	1-75

Crystal 19	15	0.2	0.15	75 (0)	1-75
Crystal 20	20	0.2	0.15	100 (0)	1-100

S3.1.5 MET-2

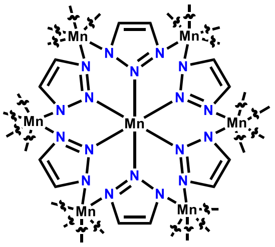

Number of crystals for which data collected: **71**
Number of datasets passing the xia2.dials processing stage (indexing, integration): **37**
Maximum number of mergeable datasets before filtering: **35**
Filtering method: **$\Delta CC_{1/2}$**
Number of datasets removed using $\Delta CC_{1/2}$: **10**
Number of data sets combined in final merged data set: **25**

***xia2.dials* commands:** integrate.min_spots.overall=10, integrate.min_spots.per_degree=0, unit_cell=18.5,18.5,18.5,90,90,90, space_group=Fd-3m, d_max=120, small_molecule=True, trust_beam_centre=True, failover=True, keep_all_reflections=False, remove_blanks=True, resolution_range=2.113-2.047, resolution_range=1.820-1.777, resolution_range=1.281-1.266, resolution_range=1.093-1.080, resolution_range=1.044-1.035, resolution_range=0.905-0.897, resolution_range=0.830-0.823, resolution_range=0.807-0.802

***xia2.multiplex* commands:** filtering.method=deltacchalf, deltacchalf.stdcutoff=3, unit_cell_clustering.threshold=20, unit_cell=18,18,18,90,90,90, resolution.d_min=0.85, min_completeness=0.95, symmetry.cosym.space_group=Fd-3m, symmetry.space_group=Fd-3m

Table S6. Experimental details for individual rotation-MCXRD data collections for **MET-2**.

Crystal number	Total crystal rotation (°)	Rotation per diffraction image (°)	Exposure per image (s)	Number of images merged (omitted)	Images used
Crystal 1	15	0.1	0.2	150 (0)	1-150
Crystal 2	15	0.1	0.5	137 (13)	1-137
Crystal 3	15	0.1	0.5	106 (44)	2-107
Crystal 4	10	0.1	0.3	100 (0)	1-100
Crystal 5	15	0.1	0.3	150 (0)	1-150
Crystal 6	10	0.1	0.3	98 (2)	2-99
Crystal 7	15	0.1	0.3	148 (2)	2-149
Crystal 8	15	0.1	0.3	148 (2)	1-148
Crystal 9	15	0.1	0.3	134 (16)	2-135
Crystal 10	15	0.1	0.25	149 (1)	1-149
Crystal 11	15	0.1	0.25	135 (15)	1-135
Crystal 12	15	0.1	0.3	150 (0)	1-150
Crystal 13	15	0.1	0.3	149 (1)	1-149
Crystal 14	15	0.1	0.3	139 (11)	1-139
Crystal 15	15	0.1	0.3	117 (33)	1-117
Crystal 16	15	0.1	0.3	148 (2)	1-148
Crystal 17	15	0.1	0.3	148 (2)	1-148
Crystal 18	15	0.1	0.3	145 (5)	1-145
Crystal 19	15	0.1	0.3	148 (2)	2-149
Crystal 20	15	0.1	0.3	146 (4)	5-150
Crystal 21	15	0.1	0.3	150 (0)	1-150
Crystal 22	15	0.1	0.3	149 (1)	1-149
Crystal 23	15	0.1	0.3	150 (0)	1-150
Crystal 24	5	0.1	0.5	50 (0)	1-50
Crystal 25	5	0.1	0.5	50 (0)	1-50

S3.1.6 MOF-919(Sc/Cu) – rotation-MCXRD

Number of conducted crystal collections: **117**
Number of datasets passing the xia2.dials processing stage (indexing, integration): **78**
Maximum number of mergeable datasets before filtering: **67**
Filtering method: **$\Delta CC_{1/2}$**
Number of datasets removed using $\Delta CC_{1/2}$: **28**
Number of datasets combined in final merged data set: **39**

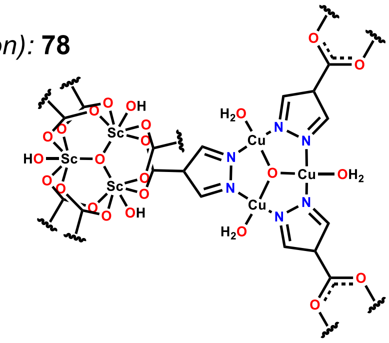

***xia2.dials* commands for crystals 1-4:** small_molecule=True, keep_all_reflections=False, d_max=120, failover=True, resolution_range=2.113,2.047, resolution_range=1.820,1.777, resolution_range=1.281,1.266, resolution_range=1.093,1.080, resolution_range=1.044,1.035, resolution_range=0.905,0.897, resolution_range=0.830,0.823, resolution_range=0.807,0.802

***xia2.dials* commands for crystals 5-39:** small_molecule=True, keep_all_reflections=False, d_max=120, failover=True, remove_blanks=True, space_group=Fd-3m, unit_cell=113,113,113,90,90,90, resolution_range=2.113,2.047, resolution_range=1.820,1.777, resolution_range=1.281,1.266, resolution_range=1.093,1.080, resolution_range=1.044,1.035, resolution_range=0.905,0.897, resolution_range=0.830,0.823, resolution_range=0.807,0.802

***xia2.multiplex* commands:** filtering.method=deltacchalf, deltacchalf.stdcutoff=3, unit_cell_clustering.threshold=20, resolution.d_min=1.15, min_completeness=0.95, symmetry.cosym.space_group=Fd-3m, symmetry.space_group=Fd-3m, resolution.space_group=Fd-3m

Table S7. Experimental details for individual rotation-MCXRD data collections for **MOF-919(Sc/Cu)**.

Crystal number	Total crystal rotation (°)	Rotation per diffraction image (°)	Exposure per image (s)	Number of images merged (omitted)	Images used	Total X-ray dose, DWD (MGy)
1	2	0.1	2	20 (0)	1-20	44.0
2	3	0.1	1.5	30 (0)	1-30	49.5
3	3	0.1	1.5	30 (0)	1-30	49.5
4	3	0.1	1.5	30 (0)	1-30	49.5
5	5	0.1	1.5	50 (0)	1-50	82.5
6	5	0.1	2	50 (0)	1-50	110.0
7	2	0.1	2	20 (0)	1-20	44.0
8	2	0.1	2	20 (0)	1-20	44.0
9	2	0.1	2	20 (0)	1-20	44.0
10	2	0.1	2	20 (0)	1-20	44.0
11	2	0.1	2	20 (0)	1-20	44.0
12	2	0.1	2	20 (0)	1-20	44.0
13	2	0.1	2	20 (0)	1-20	44.0
14	2	0.1	2	20 (0)	1-20	44.0
15	2	0.1	2	20 (0)	1-20	44.0
16	2	0.1	2	20 (0)	1-20	44.0
17	2	0.1	2	20 (0)	1-20	44.0
18	2	0.1	2	20 (0)	1-20	44.0
19	2	0.1	2	20 (0)	1-20	44.0
20	2	0.1	2	20 (0)	1-20	44.0
21	2	0.1	2	20 (0)	1-20	44.0
22	2	0.1	2	20 (0)	1-20	44.0
23	2	0.1	2	20 (0)	1-20	44.0
24	2	0.1	2	20 (0)	1-20	44.0
25	2	0.1	2	20 (0)	1-20	44.0
26	2	0.1	2	20 (0)	1-20	44.0
27	2	0.1	2	20 (0)	1-20	44.0
28	2	0.1	2	20 (0)	1-20	44.0
29	2	0.1	2	20 (0)	1-20	44.0
30	2	0.1	2	20 (0)	1-20	44.0
31	2	0.1	2	20 (0)	1-20	44.0
32	2	0.1	2	20 (0)	1-20	44.0
33	2	0.1	2	20 (0)	1-20	44.0
34	2	0.1	2	20 (0)	1-20	44.0
35	1	0.1	3	10 (0)	1-10	33.0
36	1	0.1	3	10 (0)	1-10	33.0
37	1	0.1	2.5	10 (0)	1-10	27.5
38	1	0.1	2.5	10 (0)	1-10	27.5
39	1	0.1	2.5	10 (0)	1-10	27.5

S3.1.7 MOF-919(Sc/Cu) – stationary-MCXRD (SX)

xia2.ssx commands: space_group=Fd-3m, unit_cell=114,114,114,90,90,90, max_spot_size=200, min_spot_size=6, d_min=1.0, mask=pixels.mask. spotfinding.phil=find_spots.phil, starting_geometry=refined.expt

The mask command calls a file that contains parameters that describe a mask for the beamstop shadow and for PXRD rings arising from the Cu metal present on the cryoEM grids used for they sample mount.

The spotfinding.phil command calls a file contains spot finding parameters.

The starting_geometry=refined.expt command invokes a cyclic process of optimisation of detector parameters that improves indexing and unit cell parameter determination.

***xia2.ssx_reduce* commands:** absolute_angle_tolerance=0.5, absolute_length_tolerance=0.5, d_min=1.15

Total number of grid scans: **113**
Total number of grid boxes/images: **3023**
Total number of grid scans with exposure time 2 s: **58**
Total number of grid scans with exposure time 2.5 s: **28**
Total number of grid scans with exposure time 3 s: **23**
Total number of grid scans with exposure time 4 s: **4**
Total number of grid boxes with exposure time 2 s: **1757**
Total number of grid boxes with exposure time 2.5 s: **593**
Total number of grid boxes with exposure time 3 s: **660**
Total number of grid boxes with exposure time 4 s: **13**
Average exposure time weighted by grid box number: **2.33 s**

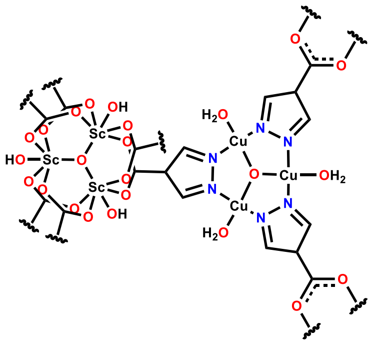

3.2 Crystallographic statistics for final merged data sets (overall and by resolution shell)

Crystallographic merging statistics were calculated using *dials* programs.[S11,S12]

S3.2.1 PCN-260(Sc)

Table S8. Overall crystallographic statistics for **PCN-260(Sc)**, including low- and high-resolution shells.

	**Overall**	**Low resolution**	**High resolution**
**Resolution, *d* (Å)**	49.21 - 0.90	49.29 - 2.44	0.92 - 0.90
**Observations**	137260	8103	2865
**Unique reflections**	25300	1429	1094
**Multiplicity**	5.4	5.7	2.6
**Completeness**	100.00%	100.00%	89.75%
**Mean $I/\sigma(I)$**	6.0	17.8	0.6
**R_{meas}**	0.203	0.108	2.238
**R_{pim}**	0.083	0.045	1.244
**$CC_{1/2}$**	0.989	0.993	0.221

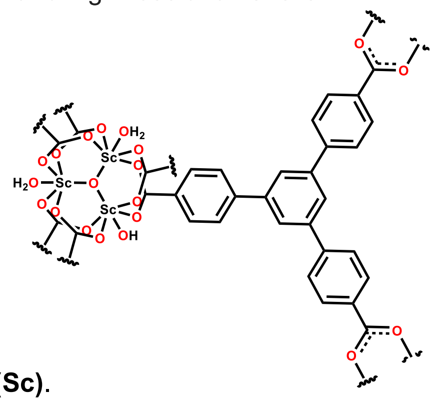

Table S9. Overall crystallographic statistics by resolution shell for **PCN-260(Sc)**.

**Resolution (Å)**	***N*(obs)**	***N*(unique)**	**Multiplicity**	**Completeness (%)**	**Mean *I***	**Mean $I/\sigma(I)$**	**R_{meas}**	**R_{pim}**	**$CC_{1/2}$**
49.29 - 2.44	8103	1429	5.7	100	474.3	17.8	0.108	0.045	0.993
2.44 - 1.94	7999	1330	6.0	100	189.2	16.2	0.119	0.048	0.991
1.94 - 1.69	8020	1296	6.2	100	90.9	13.4	0.152	0.06	0.983
1.69 - 1.54	8307	1311	6.3	100	79.7	12.7	0.161	0.063	0.987
1.54 - 1.43	8101	1296	6.3	100	58.3	10.3	0.200	0.08	0.986
1.43 - 1.34	7920	1264	6.3	100	36.9	8.3	0.258	0.102	0.980
1.34 - 1.28	8162	1294	6.3	100	21.3	5.8	0.382	0.15	0.971
1.28 - 1.22	7990	1282	6.2	100	16.5	4.8	0.482	0.191	0.941
1.22 - 1.17	7912	1253	6.3	100	14.1	4.1	0.578	0.227	0.921
1.17 - 1.13	8105	1277	6.4	100	14.1	4.0	0.577	0.226	0.951
1.13 - 1.10	7992	1264	6.3	100	12.2	3.5	0.661	0.258	0.920
1.10 - 1.07	8094	1267	6.4	100	10.6	3.2	0.731	0.285	0.897
1.07 - 1.04	7985	1260	6.3	100	9.4	2.9	0.860	0.335	0.889
1.04 - 1.01	7149	1273	5.6	100	8.7	2.6	0.868	0.358	0.839
1.01 - 0.99	5723	1255	4.6	100	6.2	1.8	1.170	0.529	0.655
0.99 - 0.97	5081	1240	4.1	100	5.5	1.5	1.204	0.566	0.535
0.97 - 0.95	4516	1256	3.6	100	4.4	1.2	1.470	0.73	0.357
0.95 - 0.93	3795	1191	3.2	99.33	3.8	0.9	1.664	0.856	0.324
0.93 - 0.92	3441	1168	3.0	95.89	3.4	0.8	1.910	1.011	0.251
0.92 - 0.90	2865	1094	2.6	89.75	2.5	0.6	2.238	1.244	0.221

S3.2.2 MIL-88(Cr)-1,4-NDC

Table S10. Overall crystallographic statistics for **MIL-88(Cr)-1,4-NDC**, including low- and high-resolution shells.

	Overall	Low resolution	High resolution
**Resolution, *d* (Å)**	12.23 - 0.74	12.23 - 2.02	0.76 - 0.74
**Observations**	20569	1656	38
**Unique reflections**	1422	104	29
**Multiplicity**	14.5	15.9	1.3
**Completeness**	97.87%	100.00%	45.31%
**Mean *I*/σ(*I*)**	21.2	58.4	0.6
R_{meas}	0.066	0.047	0.338
R_{pim}	0.016	0.013	0.239
$CC_{1/2}$	0.999	0.999	0.944

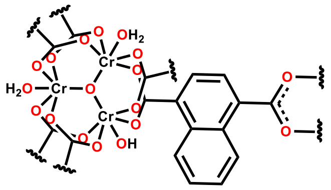

Table S11. Overall crystallographic merging statistics by resolution shell for **MIL-88(Cr)-1,4-NDC**.

Resolution (Å)	*N*(obs)	*N*(unique)	Multiplicity	Completeness (%)	Mean *I*	Mean *I*/σ(*I*)	R_{meas}	R_{pim}	$CC_{1/2}$
12.23 - 2.02	1656	104	15.9	100	11185.4	58.4	0.047	0.013	0.999
2.02 - 1.60	1725	88	19.6	100	4030.6	53.4	0.051	0.012	1.000
1.60 - 1.40	1689	81	20.9	100	3237.7	51.4	0.058	0.013	0.999
1.40 - 1.27	1683	81	20.8	100	1606.5	42.7	0.069	0.015	0.999
1.27 - 1.18	1640	74	22.2	100	847.0	31.7	0.102	0.022	0.994
1.18 - 1.11	1669	79	21.1	100	412.3	21.1	0.128	0.026	0.998
1.11 - 1.06	1716	81	21.2	100	439.9	20.2	0.14	0.03	0.999
1.06 - 1.01	1640	70	23.4	100	411.1	19.7	0.133	0.027	0.994
1.01 - 0.97	1744	82	21.3	100	241.8	15.3	0.177	0.036	0.996
0.97 - 0.94	1175	71	16.6	100	265.9	13.9	0.193	0.045	0.967
0.94 - 0.91	1043	76	13.7	100	191.1	9.6	0.179	0.046	0.991
0.91 - 0.88	828	69	12.0	100	119.7	7.1	0.26	0.073	0.979
0.88 - 0.86	650	75	8.7	98.68	120.8	6.3	0.215	0.068	0.982
0.86 - 0.84	516	70	7.4	100	127.7	4.5	0.229	0.079	0.981
0.84 - 0.82	410	69	5.9	100	74.2	2.9	0.345	0.133	0.965
0.82 - 0.80	322	70	4.6	92.11	59.4	2.2	0.367	0.16	0.896
0.80 - 0.79	215	57	3.8	90.48	41.3	1.6	1.47	0.633	0.803
0.79 - 0.77	136	56	2.4	76.71	27.9	0.8	1.089	0.587	0.877
0.77 - 0.76	74	40	1.9	57.97	33.9	0.7	0.45	0.277	0.678
0.76 - 0.74	38	29	1.3	45.31	33.7	0.6	0.338	0.239	0.944

S3.2.3 UiO-66

Table S12. Overall crystallographic statistics for **UiO-66**, including low- and high-resolution shells.

	Overall	Low resolution	High resolution
**Resolution, *d* (Å)**	11.99 - 0.80	11.99 - 2.17	0.81 - 0.80
**Observations**	14050	920	201
**Unique reflections**	515	40	22
**Multiplicity**	27.3	23	9.1
**Completeness**	100.00%	100.00%	100.00%
**Mean *I*/σ(*I*)**	49.3	53.4	22.3
R_{meas}	0.053	0.06	0.081
R_{pim}	0.055	0.062	0.086
$CC_{1/2}$	0.997	0.998	0.998

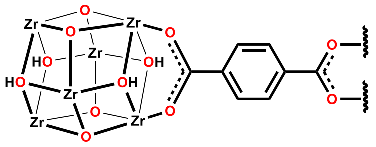

Table S13. Overall crystallographic merging statistics by resolution shell for **UiO-66**.

Resolution (Å)	*N*(obs)	*N*(unique)	Multiplicity	Completeness (%)	Mean *I*	Mean *I*/σ(*I*)	R_{meas}	R_{pim}	$CC_{1/2}$
11.99 - 2.17	920	40	23.0	100	13259.9	53.4	0.062	0.016	0.998
2.17 - 1.72	847	31	27.3	100	9671	56.6	0.049	0.011	0.991
1.72 - 1.51	851	27	31.5	100	6651.1	64.8	0.048	0.009	0.999
1.51 - 1.37	854	29	29.5	100	5295.7	59.3	0.048	0.009	0.999
1.37 - 1.27	792	25	31.7	100	2720.3	55.4	0.054	0.01	1.000
1.27 - 1.20	935	29	32.2	100	1648	54.7	0.054	0.01	1.000
1.20 - 1.14	732	23	31.8	100	3397	53.7	0.055	0.011	0.999
1.14 - 1.09	898	28	32.1	100	2660.4	60.0	0.048	0.008	1.000
1.09 - 1.05	609	19	32.1	100	3238.7	61.6	0.053	0.01	1.000
1.05 - 1.01	972	29	33.5	100	1764.2	51.9	0.055	0.009	0.999

1.01 - 0.98	715	21	34.1	100	1913.2	54.5	0.059	0.011	0.999
0.98 - 0.95	817	27	30.3	100	1504.9	45.6	0.056	0.011	1.000
0.95 - 0.92	848	25	33.9	100	1380.7	50.4	0.064	0.011	0.999
0.92 - 0.90	646	21	30.8	100	1816.4	53.8	0.062	0.012	0.999
0.90 - 0.88	803	25	32.1	100	979.2	47.6	0.067	0.012	0.999
0.88 - 0.86	579	23	25.2	100	1550.3	38.2	0.061	0.012	0.999
0.86 - 0.85	517	28	18.5	100	829.3	33.3	0.069	0.015	0.999
0.85 - 0.83	266	19	14.0	100	921.9	30.4	0.07	0.018	0.999
0.83 - 0.81	248	24	10.3	100	1201.9	26.9	0.073	0.022	0.999
0.81 - 0.80	201	22	9.1	100	612.4	22.3	0.086	0.027	0.998

S3.2.4 UiO-66-MoO_4

Table S14. Overall crystallographic statistics for **UiO-66-MoO_4**, including low- and high-resolution shells.

	Overall	Low resolution	High resolution
**Resolution, *d* (Å)**	11.97 - 0.80	11.98 - 2.17	0.81 - 0.80
**Observations**	24515	1526	311
**Unique reflections**	515	40	22
**Multiplicity**	47.6	38.1	14.1
**Completeness**	100.00%	100.00%	100.00%
**Mean $I/\sigma(I)$**	57.5	82.1	14.9
**R_{meas}**	0.064	0.056	0.164
**R_{pim}**	0.010	0.013	0.041
**$CC_{1/2}$**	0.999	0.998	0.999

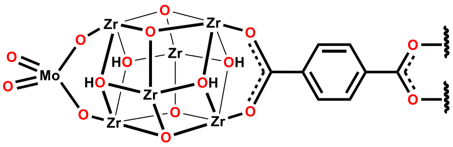

Table S15. Overall crystallographic merging statistics by resolution shell for **UiO-66**.

Resolution (Å)	*N*(obs)	*N*(unique)	Multiplicity	Completeness (%)	Mean *I*	Mean $I/\sigma(I)$	R_{meas}	R_{pim}	$CC_{1/2}$
11.98 - 2.17	1526	40	38.2	100	2909.7	82.1	0.056	0.013	0.998
2.17 - 1.72	1433	31	46.2	100	1759.5	85.1	0.05	0.008	0.999
1.72 - 1.50	1481	27	54.9	100	1251.3	97.9	0.049	0.007	1.000
1.50 - 1.37	1520	29	52.4	100	845.4	84.7	0.055	0.008	1.000
1.37 - 1.27	1421	25	56.8	100	406.4	66.1	0.081	0.013	0.999
1.27 - 1.19	1591	29	54.9	100	234.4	57.4	0.078	0.011	1.000
1.19 - 1.14	1287	23	56.0	100	511.7	64.6	0.065	0.009	1.000
1.14 - 1.09	1555	28	55.5	100	385.8	71.3	0.069	0.009	1.000
1.09 - 1.04	1116	19	58.7	100	442.5	75.9	0.063	0.008	1.000
1.04 - 1.01	1643	29	56.7	100	222.1	56	0.083	0.01	0.999
1.01 - 0.98	1246	21	59.3	100	238.6	52.6	0.088	0.012	1.000
0.98 - 0.95	1468	27	54.4	100	180	45.3	0.095	0.013	1.000
0.95 - 0.92	1550	25	62.0	100	159.4	51.1	0.101	0.013	0.999
0.92 - 0.90	1052	21	50.1	100	217.4	51.3	0.101	0.015	0.999
0.90 - 0.88	1429	25	57.2	100	110.9	44.1	0.119	0.016	0.999
0.88 - 0.86	1133	23	49.3	100	171.9	37.4	0.094	0.014	0.999
0.86 - 0.84	804	28	28.7	100	88.1	25	0.117	0.022	0.998
0.84 - 0.83	494	19	26.0	100	98.7	25.8	0.127	0.026	0.999
0.83 - 0.81	455	24	19.0	100	118.6	24	0.131	0.03	0.999
0.81 - 0.80	311	22	14.1	100	60.7	14.9	0.164	0.041	0.999

S3.2.5 MET-2

Table S16. Overall crystallographic statistics for **MET-2**, including low- and high-resolution shells.

	Overall	Low resolution	High resolution
**Resolution, *d* (Å)**	10.48 - 0.85	10.48 - 2.31	0.87 - 0.85
**Observations**	5742	867	84
**Unique reflections**	288	25	11
**Multiplicity**	19.9	34.7	7.6
**Completeness**	100.00%	100.00%	91.67%
**Mean $I/\sigma(I)$**	17.2	36.0	5.3
**R_{meas}**	0.139	0.117	0.951
**R_{pim}**	0.027	0.022	0.311
**$CC_{1/2}$**	0.992	0.992	0.952

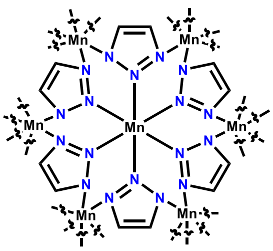

Table S17. Overall crystallographic merging statistics by resolution shell for **MET-2**.

Resolution (Å)	*N*(obs)	*N*(unique)	Multiplicity	Completeness (%)	Mean *I*	Mean *I*/σ(*I*)	R_{meas}	R_{pim}	$CC_{1/2}$
10.48 - 2.31	867	25	34.7	100	240.6	36	0.117	0.022	0.992
2.31 - 1.84	459	13	35.3	86.67	135.4	35.2	0.12	0.024	0.990
1.84 - 1.60	527	15	35.1	100	59	26.3	0.131	0.023	0.999
1.60 - 1.46	729	18	40.5	100	69.9	32.6	0.157	0.028	1.000
1.46 - 1.35	753	17	44.3	100	51.1	32.2	0.153	0.024	0.999
1.35 - 1.27	382	12	31.8	100	29.8	18.3	0.204	0.045	0.997
1.27 - 1.21	374	16	23.4	100	17.1	15.2	0.222	0.042	0.997
1.21 - 1.16	274	15	18.3	100	15.0	18.4	0.178	0.039	0.948
1.16 - 1.11	275	15	18.3	100	8.5	11.1	0.337	0.085	0.966
1.11 - 1.08	81	11	7.4	84.62	8.7	9.1	0.317	0.120	0.978
1.08 - 1.04	110	15	7.3	100	14.6	11.2	0.268	0.095	0.971
1.04 - 1.01	106	9	11.8	90	13.9	10.9	0.229	0.065	0.999
1.01 - 0.99	154	18	8.6	100	10.6	10.9	0.239	0.071	0.973
0.99 - 0.96	118	15	7.9	100	3.9	6.5	0.43	0.147	0.956
0.96 - 0.94	134	13	10.3	100	4.8	10.8	0.342	0.099	0.961
0.94 - 0.92	74	11	6.7	100	4.8	7.0	0.274	0.126	0.924
0.92 - 0.90	71	13	5.5	81.25	3.4	3.8	0.49	0.188	0.983
0.90 - 0.88	88	15	5.9	100	3.8	6.2	0.434	0.158	0.963
0.88 - 0.87	82	11	7.5	100	3.4	7.9	0.394	0.134	0.962
0.87 - 0.85	84	11	7.6	91.67	1.3	5.3	0.951	0.311	0.952

S3.2.6 MOF-919(Sc/Cu) – rotation-MCXRD

Table S18. Overall crystallographic merging statistics for **MOF-919(Sc/Cu)** – rotation-MCXRD, including low- and high-resolution shells.

	Overall	Low resolution	High resolution
**Resolution, *d* (Å)**	66.02 - 1.15	66.13 - 3.12	1.17 - 1.15
**Observations**	321556	21336	7952
**Unique reflections**	22616	1322	1121
**Multiplicity**	14.2	16.1	7.1
**Completeness**	98.97%	100.00%	100.00%
**Mean *I*/σ(*I*)**	4.2	14.1	0.4
R_{meas}	0.358	0.26	4.36
R_{pim}	0.09	0.065	1.611
$CC_{1/2}$	0.979	0.98	0.199

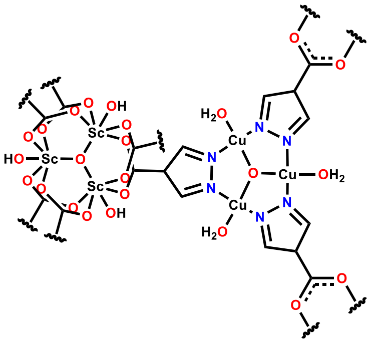

Table S19. Crystallographic merging statistics by resolution shell for **MOF-919(Sc/Cu)** – rotation-MCXRD.

Resolution (Å)	*N*(obs)	*N*(unique)	Multiplicity	Completeness (%)	Mean *I*	Mean *I*/σ(*I*)	R_{meas}	R_{pim}	$CC_{1/2}$
66.13 - 3.12	21336	1322	16.1	100	43.1	14.1	0.260	0.065	0.980
3.12 - 2.48	21107	1221	17.3	100	24.5	11.9	0.266	0.064	0.980
2.48 - 2.16	21754	1198	18.2	100	12.7	9.1	0.309	0.072	0.984
2.16 - 1.97	13447	926	14.5	79.9	12.7	7.1	0.312	0.079	0.976
1.97 - 1.83	21562	1188	18.2	100	7.3	6.4	0.369	0.087	0.985
1.83 - 1.72	11460	899	12.8	79.35	5.5	4.4	0.398	0.105	0.814
1.72 - 1.63	20478	1162	17.6	100	5.5	5.1	0.418	0.101	0.947
1.63 - 1.56	20228	1160	17.4	100	4.4	4.4	0.432	0.104	0.961
1.56 - 1.50	19744	1159	17.0	100	3.3	3.4	0.527	0.130	0.978
1.50 - 1.45	19754	1161	17.0	100	2.8	3.2	0.553	0.133	0.941
1.45 - 1.40	19204	1147	16.7	100	2.7	3.0	0.590	0.144	0.975
1.40 - 1.36	18881	1143	16.5	100	1.7	2.2	0.805	0.196	0.914
1.36 - 1.33	17674	1153	15.3	100	1.4	1.8	0.923	0.233	0.862
1.33 - 1.30	15583	1132	13.8	100	1.2	1.6	1.124	0.300	0.827
1.30 - 1.27	8394	982	8.6	87.99	1.2	0.8	2.389	0.808	0.246
1.27 - 1.24	10323	1128	9.15	99.82	0.6	0.7	2.019	0.652	0.302
1.24 - 1.21	13014	1143	11.4	100	0.5	0.8	2.224	0.644	0.675
1.21 - 1.19	10522	1124	9.4	100	0.4	0.6	2.654	0.859	0.497
1.19 - 1.17	9139	1147	8.0	100	0.4	0.5	3.454	1.193	0.436
1.17 - 1.15	7952	1121	7.0	100	0.3	0.4	4.36	1.611	0.199

S3.2.7 **MOF-919(Sc/Cu)** – stationary-MCXRD (SX)

Table S20. Overall crystallographic merging statistics for **MOF-919(Sc/Cu)** – stationary-MCXRD (SX), including low- and high-resolution shells.

	Overall	Low resolution	High resolution
**Resolution, *d* (Å)**	40.46 - 1.15	40.48 - 3.12	1.17 - 1.15
**Observations**	1238100	147821	28134
**Unique reflections**	22346	1269	1149
**Multiplicity**	55.4	116.5	24.5
**Completeness**	97.52%	100.00%	100.00%
**Mean $I/\sigma(I)$**	15.5	73.6	0.8
R_{meas}	0.555	0.336	12.118
R_{pim}	0.067	0.034	2.423
R_{split}	0.092	0.037	1.468
$CC_{1/2}$	0.994	0.998	0.222

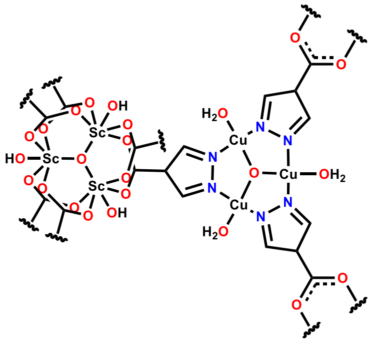

Table S21. Crystallographic merging statistics by resolution shell for **MOF-919(Sc/Cu)** – stationary-MCXRD (SX).

Resolution (Å)	*N*(obs)	*N*(unique)	Multiplicity	Completeness (%)	Mean *I*	Mean $I/\sigma(I)$	R_{meas}	R_{pim}	R_{split}	$CC_{1/2}$
40.48 - 3.12	147821	1269	116.49	100	366.2	73.6	0.336	0.034	0.037	0.998
3.12 - 2.48	113039	1196	94.51	100	195.2	54.9	0.375	0.040	0.043	0.979
2.48 - 2.16	96957	1166	83.15	100	95.5	34.5	0.495	0.055	0.060	0.995
2.16 - 1.97	61833	1020	60.62	88.24	101.8	25.8	0.575	0.075	0.143	0.929
1.97 - 1.83	75941	1156	65.69	100	50.9	19.8	0.725	0.090	0.096	0.988
1.83 - 1.72	50820	979	51.91	85.2	41.8	14.2	0.801	0.106	0.207	0.857
1.72 - 1.63	76536	1136	67.37	100	38.4	16.5	0.801	0.098	0.114	0.990
1.63 - 1.56	77770	1128	68.95	100	28.8	13.5	0.943	0.114	0.122	0.980
1.56 - 1.50	72765	1143	63.66	100	20.3	9.4	1.283	0.161	0.148	0.986
1.50 - 1.45	66054	1136	58.15	100	17.3	8.5	1.349	0.177	0.171	0.970
1.45 - 1.40	60716	1136	53.45	100	15.7	7.3	1.464	0.200	0.178	0.983
1.40 - 1.36	55245	1117	49.46	100	9.6	4.9	2.103	0.297	0.250	0.917
1.36 - 1.33	49420	1144	43.2	100	7.7	3.9	2.521	0.380	0.313	0.912
1.33 - 1.30	47233	1130	41.8	99.91	6.7	3.1	3.215	0.495	0.372	0.902
1.30 - 1.27	15190	861	17.64	76.53	2.7	1.1	8.106	1.743	2.495	0.086
1.27 - 1.24	37720	1127	33.47	100	3.1	1.5	6.959	1.179	0.825	0.615
1.24 - 1.21	38402	1125	34.14	100	2.9	1.4	7.044	1.194	0.796	0.647
1.21 - 1.19	35358	1112	31.8	100	2.4	1.2	8.201	1.439	0.987	0.446
1.19 - 1.17	31146	1116	27.91	100	2.1	1.0	10.135	1.898	1.194	0.403
1.17 - 1.15	28134	1149	24.49	100	1.7	0.8	12.118	2.423	1.468	0.222

S3.3 Crystallographic statistics for data sets from individual crystals

Crystallographic information is provided in Tables S22-S27 for the rotation-MCXRD studies from data processing by *xia2.dials*.

For **MIL-88(Cr)-1,4-NDC**, **UiO-66**, **UiO-66-MoO$_4$**, **MET-2** and **MIL-919(Sc/Cu)**, crystallographic statistics are reported for the data sets from individual crystals that were combined in the final merged data set. For **PCN-260(Sc)** (Table S22), data sets presented in bold font are those used in the final merged data set and data sets presented in italic font passed the *xia2.dials* processing stage (*i.e.* were indexed and integrated) but were omitted from the final merged data set due to unit cell mismatch or high R_{pim}. All statistics reported for data from individual crystals have been determined by application of the final resolution cutoff and unit cell parameters used for the corresponding overall merged data set.

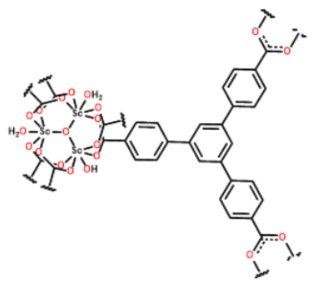

Table S22. Crystallographic statistics for individual data sets of **PCN-260(Sc)**.

Crystal number / [collection number]	*a* (Å)	*b* (Å)	*c* (Å)	*V* (Å^3)	R_{meas}	R_{pim}	$CC_{1/2}$	$I/\sigma(I)$	Completeness (%)	Multiplicity	Total reflns	Unique reflns
**Crystal 1 [14]**	**36.2961(12)**	**18.72111(18)**	**49.1599(9)**	**33404.3(9)**	**0.188**	**0.11**	**0.98**	**3.90**	**74.9**	**1.80**	**40074**	**18200**
**Crystal 2 [17]**	**36.2453(3)**	**18.7359(7)**	**49.2500(3)**	**33445.1(12)**	**0.119**	**0.075**	**0.993**	**3.20**	**56.9**	**1.60**	**24287**	**13850**
**Crystal 3 [18]**	**36.2561(10)**	**18.7356(2)**	**49.2493(5)**	**33454.0(7)**	**0.091**	**0.057**	**0.998**	**7.40**	**60.1**	**2.50**	**24058**	**14626**
**Crystal 4 [25]**	**36.2416(3)**	**18.72402(14)**	**49.1785(15)**	**33372.0(9)**	**0.229**	**0.129**	**0.986**	**2.30**	**79.2**	**2.40**	**48562**	**19249**
Crystal [1]	36.1939(5)	18.69514(16)	49.2148(18)	33301.30(11)	0.459	0.263	0.727	1.60	68.1	2.40	39654	16505
Crystal [2]	36.1338(10)	18.6524(4)	49.113(12)	33101(8)	2.344	1.382	0.542	0.80	68	1.50	40309	16604
Crystal [3]	36.2579(4)	18.7232(3)	49.257(3)	33438.9(17)	0.41	0.28	0.763	3.00	43.5	4.20	16069	10618
Crystal [5]	36.425(2)	18.7034(6)	49.2864(17)	33577.5(18)	6.324	3.012	0.055	1.30	77.9	1.40	80197	19105
Crystal [9]	36.2542(6)	18.7206(3)	49.1822(7)	33380.0(5)	1.106	0.76	0.769	1.10	68.2	1.70	23849	16577
Crystal [13]	36.2496(8)	18.7086(3)	49.1503(9)	33332.7(6)	0.871	0.551	0.865	1.90	76.2	2.50	32001	18507
Crystal [16]	36.1845(9)	18.7124(9)	49.140(7)	33273(4)	4.327	2.359	0.193	0.40	78.3	2.70	47959	18981
Crystal [22]	36.2375(3)	18.72439(13)	49.2318(16)	33405.0(9)	0.378	0.205	0.986	5.00	61.9	2.60	40393	15059
Crystal [23]	36.2537(6)	18.7391(4)	49.286(3)	33482.8(19)	0.933	0.528	0.908	0.60	76.5	2.70	48293	18613
Crystal [26]	36.2315(10)	18.7334(12)	49.2375(15)	33419.40(19)	1.488	0.777	0.93	0.60	73.2	2.30	48490	17817
Crystal [28]	36.2180(4)	18.7604(9)	49.1582(9)	33401.30(14)	0.334	0.186	0.991	3.00	57.3	3.30	32174	13967
Crystal [29]	36.2629(6)	18.73214(15)	49.2336(7)	33443.5(6)	8.613	4.922	0.028	1.20	95.4	3.80	77715	23235
Crystal [30]	36.2863(8)	18.6992(3)	49.1873(11)	33374.7(8)	2.39	1.247	0.56	0.90	96.4	2.70	88546	23469
Crystal [31]	36.302(6)	18.7291(9)	49.366(9)	33563(6)	10.499	5.631	0.276	0.20	73.2	2.70	48384	17959
Crystal [32]	36.2714(7)	18.7391(3)	49.2839(15)	33498.0(1)	1.061	0.566	0.906	0.70	86.4	1.50	55939	21031
Crystal [36]	36.2556(18)	18.7345(9)	49.276(15)	33469(9)	1.647	1.091	0.761	0.40	43.8	2.30	16064	10665
Crystal [40]	36.2689(4)	18.7118(3)	49.199(4)	33389(2)	0.24	0.135	0.937	1.90	56.9	3.20	32033	13751
Crystal [44]	35.753(4)	18.4009(17)	47.43(2)	31205(14)	0.982	0.504	0.707	1.60	79.9	2.00	56910	17958
Crystal [47]	36.2729(7)	18.7356(3)	49.262(5)	33478(3)	0.449	0.275	0.967	1.10	49.3	1.80	24234	12014

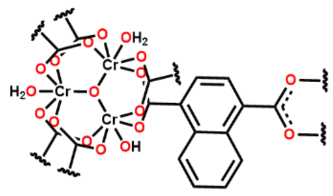

Table S23. Crystallographic statistics for individual data sets of **MIL-88B(Cr)-1,4-NDC**.

Crystal number	*a* (Å)	*c* (Å)	*V* (Å^3)	R_{meas}	R_{pim}	$CC_{1/2}$	$I/\sigma(I)$	Completeness (%)	Multiplicity	Total reflns	Unique reflns
Crystal 1	14.1525(4)	17.1846(7)	2980.82(14)	0.025	0.014	1.000	25.8	74.1	2.5	2708	1098
Crystal 2	14.1412(5)	17.1864(4)	2976.4(16)	0.122	0.068	0.976	17.6	57.3	3.1	2602	833
Crystal 3	14.1167(4)	17.1963(5)	2967.77(12)	0.042	0.027	0.997	17.2	69.4	1.9	1827	987
Crystal 4	14.1024(5)	17.2177(5)	2965.44(17)	0.048	0.02	0.998	15.9	91.2	5.4	7148	1325
Crystal 5	14.1153(3)	17.1944(3)	2966.86(9)	0.037	0.019	0.999	18.0	69.3	2.7	2755	1002
Crystal 6	14.0609(6)	17.2252(10)	2949.32(19)	0.043	0.022	0.999	18.3	76.6	3.3	3617	1089

Table S24. Crystallographic statistics for individual data sets of **UiO-66**.

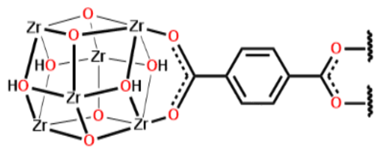

Crystal number	*a* (Å)	*V* (Å^3)	Total crystal rotation (°)	Exposure per image (s)	R_{meas}	R_{pim}	$CC_{1/2}$	$I/\sigma(I)$	Completeness (%)	Multiplicity	Total reflns	Unique reflns
Crystal 1	20.7664(4)	8955.4(5)	20	0.3	0.028	0.016	0.999	42.7	90.3	3	1403	464
Crystal 2	20.7648(3)	8953.3(4)	20	0.3	0.032	0.018	0.998	41.4	88	3.1	1408	453
Crystal 3	20.7723(3)	8963.0(3)	20	0.3	0.037	0.019	0.998	39.2	70.3	4	1438	362
Crystal 4	20.7573(3)	8943.6(4)	20	0.3	0.04	0.021	0.998	33	88.3	3.2	1426	452
Crystal 5	20.76736(19)	8956.6(2)	20	0.3	0.039	0.02	0.999	33.7	90	3.1	1439	460
Crystal 6	20.7722(4)	8962.9(5)	20	0.3	0.041	0.02	0.999	24.3	74.8	3.7	1405	383
Crystal 7	20.76942(16)	8959.3(2)	20	0.3	0.044	0.022	0.997	31.1	74.6	3.8	1455	381
Crystal 8	20.7617(5)	8949.3(6)	20	0.3	0.051	0.027	0.998	22.8	88.7	3.1	1432	457
Crystal 9	20.7669(7)	8956.0(8)	20	0.3	0.136	0.092	0.929	32	90.9	2.4	1112	468
Crystal 10	20.7615(2)	8949.0(3)	20	0.3	0.069	0.035	0.995	22.7	75.7	3.7	1444	390

Table S25. Crystallographic statistics for individual data sets of **UiO-66-MoO$_4$**.

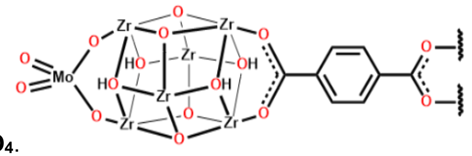

Crystal number	*a* (Å)	Volume (Å^3)	Total crystal rotation (°)	Exposure per image (s)	R_{meas}	R_{pim}	$CC_{1/2}$	$I/\sigma(I)$	Completeness (%)	Multiplicity	Total reflns	Unique reflns
Crystal 1	20.7463(4)	8929.4(5)	15	0.15	0.036	0.021	0.999	29.3	85.2	2.4	1032	438
Crystal 2	20.7367(4)	8917.0(5)	20	0.15	0.044	0.022	0.998	29.3	76.2	3.7	1438	388
Crystal 3	20.7365(4)	8916.7(5)	15	0.15	0.049	0.028	0.997	23.2	88.7	2.4	1099	455
Crystal 4	20.7400(3)	8921.3(4)	20	0.15	0.051	0.027	0.996	26.5	84.8	3.3	1435	434
Crystal 5	20.7333(4)	8912.6(5)	20	0.15	0.051	0.027	0.999	22.6	92.0	3.1	1450	470
Crystal 6	20.7442(2)	8926.7(3)	15	0.15	0.053	0.029	0.998	25.1	80.3	2.6	1067	411
Crystal 7	20.7446(4)	8927.2(5)	15	0.15	0.054	0.03	0.998	21.7	76.2	2.7	1066	390
Crystal 8	20.7388(2)	8919.7(3)	20	0.15	0.054	0.029	0.997	21.3	90.6	3.1	1460	465
Crystal 9	20.7362(4)	8916.4(6)	15	0.15	0.059	0.034	0.998	15.6	88.6	2.3	1051	453
Crystal 10	20.7359(4)	8916.0(6)	20	0.15	0.056	0.03	0.998	21.3	86.5	3.2	1439	443
Crystal 11	20.7391(2)	8920.1(3)	15	0.15	0.063	0.035	0.998	16.9	84.1	2.7	1132	417
Crystal 12	20.7345(3)	8914.2(3)	20	0.15	0.062	0.032	0.996	18.1	89.8	3.2	1469	460
Crystal 13	20.7466(4)	8929.8(5)	15	0.15	0.067	0.039	0.997	17.7	86.2	2.5	1087	443
Crystal 14	20.7427(3)	8924.8(4)	15	0.15	0.064	0.036	0.996	18.3	82.3	2.5	1076	424
Crystal 15	20.7349(2)	8914.7(3)	20	0.15	0.064	0.034	0.997	16.6	91.6	3.1	1469	469
Crystal 16	20.7477(4)	8931.2(5)	15	0.15	0.069	0.04	0.991	17.9	82.0	2.6	1091	419
Crystal 17	20.7498(3)	8933.9(4)	15	0.15	0.07	0.04	0.997	15.2	81.4	2.6	1070	416
Crystal 18	20.7451(4)	8927.8(5)	15	0.15	0.071	0.042	0.998	14.4	85.6	2.5	1103	441
Crystal 19	20.7499(3)	8934.0(4)	15	0.15	0.07	0.039	0.995	19.5	71.9	2.9	1073	368
Crystal 20	20.7426(4)	8924.6(6)	20	0.15	0.07	0.036	0.996	19.2	87.1	3.2	1436	445

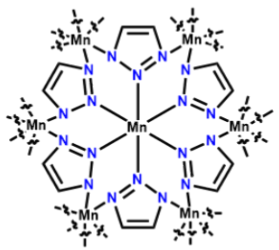

Table S26. Crystallographic statistics for individual data sets of **MET-2**.

Crystal number	*a* (Å)	*V* (Å^3)	R_{meas}	R_{pim}	$CC_{1/2}$	$I/\sigma(I)$	Completeness (%)	Multiplicity	Total reflns	Unique reflns
Crystal 1	18.156(3)	5985(3)	0.06	0.034	0.999	9.7	49.3	1.9	265	136
Crystal 2	18.1649(18)	5993.7(18)	0.063	0.036	0.999	12.6	41.7	2.1	246	116
Crystal 3	18.115(10)	5945(10)	0.067	0.04	0.994	14.5	33.5	2.0	179	91
Crystal 4	18.127(3)	5956(3)	0.045	0.027	0.999	14.2	39.0	1.7	177	106
Crystal 5	18.156(3)	5985(3)	0.07	0.041	0.999	13.1	50.9	1.9	262	138
Crystal 6	18.154(5)	5983(5)	0.039	0.025	1.000	11.7	41.1	1.5	169	113
Crystal 7	18.1797(12)	6008.4(12)	0.186	0.117	0.918	5.5	50.5	1.8	251	142
Crystal 8	18.125(7)	5955(7)	0.173	0.12	0.955	16.8	55.2	1.6	235	148
Crystal 9	18.143(2)	5972(2)	0.089	0.055	0.996	7.0	52.9	1.7	245	147
Crystal 10	18.139(4)	5968(4)	0.051	0.03	1.000	15.0	47.1	2.0	250	128
Crystal 11	18.145(3)	5975(3)	0.159	0.105	0.979	6.8	49.6	1.6	226	138
Crystal 12	18.125(3)	5955(2)	0.089	0.056	0.985	10.3	49.3	1.9	255	137
Crystal 13	18.1(4)	5930(4)	0.062	0.037	0.998	14.7	48.2	2.0	256	131
Crystal 14	18.1623(17)	5991.2(16)	0.118	0.07	0.991	6.0	51.4	1.7	247	142
Crystal 15	18.1444(19)	5973.5(18)	0.113	0.073	0.995	7.1	45.9	1.7	208	124
Crystal 16	18.156(11)	5985(11)	0.100	0.061	0.991	11.6	51.7	1.8	256	140
Crystal 17	18.149(4)	5978(4)	0.089	0.058	0.997	13.8	52.2	1.7	247	144
Crystal 18	18.258(4)	6086(4)	0.134	0.078	0.994	4.9	46.2	2.0	264	133
Crystal 19	18.181(3)	6009(3)	0.111	0.065	0.993	7.4	54.3	1.8	276	153
Crystal 20	18.154(3)	5983(3)	0.06	0.037	0.999	10.1	55.4	1.8	262	149
Crystal 21	18.167(3)	5995(3)	0.13	0.078	0.984	10.7	51.1	1.9	265	142
Crystal 22	18.127(6)	5956(6)	0.115	0.071	0.982	14.1	52.2	1.7	251	144
Crystal 23	18.118(3)	5948(3)	0.067	0.038	0.996	13.0	52.5	1.7	246	146
Crystal 24	18.111(3)	5940(3)	0.015	0.011	1.000	19.1	19.6	1.2	66	54
Crystal 25	18.1731(15)	6002.1(14)	0.044	0.026	0.997	10.4	15.8	1.9	83	44

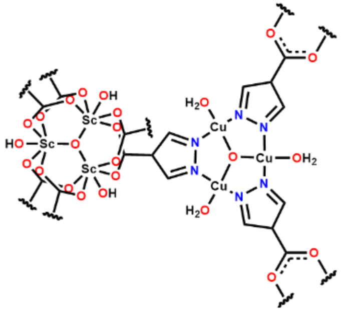

Table S27. Crystallographic statistics for individual data sets of **MOF-919(Sc/Cu) − rotation-MCXRD.**

Crystal number	*a* (Å)	*V* (Å³)	R_{meas}	R_{pim}	$CC_{1/2}$	$I/\sigma(I)$	Completeness (%)	Multiplicity	Total reflns	Unique reflns
Crystal 1	114.068(2)	1484210(80)	0.219	0.151	0.968	4.50	24.3	1.3	7184	5501
Crystal 2	113.864(3)	1476240(130)	0.376	0.255	0.941	2.70	35.3	1.3	10677	7959
Crystal 3	113.698(4)	1469800(160)	0.228	0.152	0.974	3.10	32.4	1.5	10645	7273
Crystal 4	114.365(4)	1495810(140)	0.275	0.184	0.953	2.90	33.3	1.4	10832	7601
Crystal 5	115.046(3)	1522690(130)	0.295	0.195	0.947	3.10	58.9	1.6	21416	13692
Crystal 6	115.1282(19)	1525970(80)	0.342	0.231	0.883	2.90	60.2	1.5	21563	14045
Crystal 7	114.7888(17)	1512510(70)	0.184	0.128	0.979	6.30	30.1	1.2	8256	6944
Crystal 8	114.8238(17)	1513900(70)	0.261	0.181	0.978	3.80	30.7	1.2	8268	7088
Crystal 9	114.860(2)	1515340(90)	0.171	0.118	0.975	4.80	29.7	1.2	8416	6882
Crystal 10	115.209(3)	1529190(100)	0.17	0.116	0.985	4.10	27.0	1.3	8088	6315
Crystal 11	115.058(3)	1523170(110)	0.271	0.187	0.983	2.20	28.9	1.2	7949	6733
Crystal 12	114.857(5)	1515210(190)	0.309	0.213	0.954	2.00	27.8	1.2	8015	6442
Crystal 13	114.7139(16)	1509550(60)	0.391	0.269	0.891	3.30	29.2	1.2	7825	6731
Crystal 14	115.0148(16)	1521460(60)	0.304	0.211	0.87	4.60	29.2	1.2	7904	6781
Crystal 15	114.693(2)	1508710(80)	0.233	0.16	0.962	5.00	29.7	1.2	7858	6830
Crystal 16	114.538(3)	1502620(130)	0.317	0.219	0.894	2.90	29.4	1.2	7851	6738
Crystal 17	114.780(2)	1512180(90)	0.221	0.151	0.962	3.30	28.2	1.2	7898	6524
Crystal 18	114.5864(19)	1504520(70)	0.231	0.162	0.967	6.10	28.7	1.2	7849	6585
Crystal 19	114.455(3)	1499340(100)	0.281	0.194	0.928	4.00	29.2	1.2	7947	6687
Crystal 20	114.500(2)	1501120(90)	0.287	0.200	0.934	3.50	27.5	1.2	7794	6302
Crystal 21	114.7253(16)	1510000(70)	0.179	0.125	0.967	6.40	26.6	1.3	7754	6131
Crystal 22	114.795(2)	1512760(90)	0.253	0.175	0.956	4.00	28.6	1.2	7873	6601
Crystal 23	114.866(2)	1515550(90)	0.199	0.138	0.966	3.50	30.1	1.1	7943	6968
Crystal 24	114.5003(15)	1501130(60)	0.352	0.244	0.878	4.30	28.8	1.2	7747	6616
Crystal 25	114.647(5)	1506930(210)	0.461	0.316	0.879	2.10	28.1	1.2	7937	6466
Crystal 26	113.814(5)	1474320(200)	0.332	0.228	0.945	1.80	27.7	1.2	7685	6242
Crystal 27	113.781(3)	1473040(110)	0.291	0.201	0.97	2.70	29.9	1.2	7829	6726
Crystal 28	113.755(3)	1472030(120)	0.151	0.104	0.992	3.90	29.0	1.2	7518	6523
Crystal 29	113.7161(19)	1470500(70)	0.204	0.143	0.972	4.00	29.0	1.2	7659	6504
Crystal 30	113.955(2)	1479790(90)	0.318	0.222	0.9	3.40	28.9	1.2	7645	6540
Crystal 31	113.785(2)	1473180(100)	0.354	0.248	0.951	3.20	28.6	1.2	7711	6430
Crystal 32	113.771(2)	1472650(80)	0.198	0.133	0.963	3.80	23.8	1.4	7658	5365
Crystal 33	113.8294(14)	1474900(50)	0.163	0.114	0.972	6.00	28.6	1.2	7637	6442
Crystal 34	113.893(3)	1477400(120)	0.244	0.168	0.963	2.90	28.0	1.2	7727	6308
Crystal 35	114.153(3)	1487520(140)	0.162	0.113	0.988	4.20	16.0	1.1	3964	3626
Crystal 36	113.7031(18)	1470000(70)	0.148	0.105	0.982	5.50	14.3	1.2	3864	3218
Crystal 37	114.048(3)	1483410(130)	0.424	0.299	0.853	3.30	16.1	1.1	3888	3639
Crystal 38	114.079(3)	1484630(100)	0.247	0.174	0.941	4.80	16.0	1.1	3930	3633
Crystal 39	113.902(2)	1477740(90)	0.167	0.116	0.985	4.60	13.2	1.3	3928	2972

S4. *RADDOSE-3D* X-ray dose calculations for MOF-919(Sc/Cu)

RADDOSE-3D v4.0[S34,S35] was used to calculate the spatial and temporal X-ray dose absorbed by MOF-919(Sc/Cu) crystals. *RADDOSE-3D* calculations were applied to the X-ray fluorescence scans (beamline I24) used to investigate potential radiation-induced redox activity at the Cu centres (Section S4.1), and for X-ray data collections (beamline VMXm) used for crystal structure determination (Section S4.2). Dose calculations used a crystal size of 4 x 4 x 4 µm and cubic unit cell parameters (*a* = 114.4 Å) with unit cell content set to C 6528 H 7344 N 3264 O 6528 Cu 1632 Sc 816 based on a formula unit of composition $C_{24}H_{27}N_{12}O_{24}Cu_6Sc_3$ (*i.e.* $\{Sc_3(\mu_3\text{-}O)(OH)_3\}(\mu\text{-PyC})_6\{Cu_3(\mu_3\text{-}O)(OH_2)_3\}_2$) and $Z = 272$ ($Z' = 1^5/_{12}$). Absorption coefficients were calculated using the "*SMALLMOLE*" command[S35] and a resolution of 5 pixels/µm was used. Pore contents, which are undetermined, were not included in X-ray dose calculations. Thus, the reported dose values are only considered as lower-bound estimates of the true values.

S4.1 X-Ray fluorescence dose measurements and calculations for MOF-919(Sc/Cu)

MOF-919(Sc/Cu) crystals were pipetted in DMF onto a MiTeGen MicroMesh Mylar sample mount and placed within a flow of N_2 gas at 100 K from an Oxford Cryosystems Cryostream device. Fluorescence emissions were captured using a vortex fluorescence detector at the I24 beamline. A single MOF-919(Sc/Cu) crystal was irradiated with 7 sequential copper edge scans between energies of 8949 and 9019 eV on beamline I24 with sample temperature maintained at 100 K using an Oxford Cryosystems Cryostream cooling device. An X-ray exposure time of 0.5 seconds per data point was used with 46 independent fluorescence measurements per scan during which time the crystal was not rotated. Dose calculations assumed a FWHM 9 x 9 µm Gaussian-shaped beam. The photon flux was calculated individually for each scan (1-7) using the current recorded by an X-Ray Beam Position Monitor (XBPM) situated immediately before the sample. The XBPM current was converted to a photon flux value using a calibrated 500 µm thick silicon diode and following the approach described by Owen *et al.*[S36]

The resulting average diffraction weighted dose (DWD) values[S37] for scans 1-7 were 1.00, 1.05, 1.01, 1.06, 1.00, 2.46 and 2.41 MGy, respectively. X-Ray dose on the crystal is cumulative for sequential fluorescence scans 1-7 resulting in total absorbed X-ray doses of 1.00, 2.05, 3.06, 4.12, 5.12, 7.58 and 9.99 MGy, respectively (Figure 5b).

S4.2 X-Ray dose calculations for data collections used for crystal structure determination of MOF-919(Sc/Cu) and beamline VMXm setup

For both rotation-MCXRD and stationary-MCXRD (SX) X-ray data collections on the beamline VMXm, the X-ray energy was set to 24.90 keV with a pre-calibrated photon flux value of 1.8×10^{10} ph s^{-1} with no X-ray beam attenuation. Dose calculations assumed a Gaussian-shaped beam with a FWHM of 2.8 x 3.7 µm. For the rotation-MCXRD data collections only, the "*ANGULARRESOLUTION*" parameter[S35] was invoked in *RADDOSE-3D* and set at 0.01°. For both the rotation-MCXRD and stationary-MCXRD (SX) data collections, the DWD X-ray dose was calculated at 1.1 MGy s^{-1} $image^{-1}$. For the rotation-MCXRD data collections, the DWD per crystal/dataset was calculated at 45.3 MGy. For the stationary-MCXRD (SX) measurements, the *grid-box-weighted average exposure time* was calculated at 2.3 s and used as the *ExposureTime* parameter.[S35] The DWD was calculated at 2.5 MGy $image^{-1}$.

Figure S4 shows the effects of global radiation damage for the rotation-MCXRD data collections. A concatenated plot of the data collections of the 39 crystals retained for the final merged data set of the rotation-MCXRD data collections shows the evolution of the mean $I/\sigma(I)$ of reflections on a frame-by-frame basis. Each crystal exhibits a maximum $I/\sigma(I)$ value on first frame of the data collection with a rapid decline thereafter as a consequence of global radiation damage. Thus, the concatenated plot appears as a series of spikes at the start of data collection for each crystal.

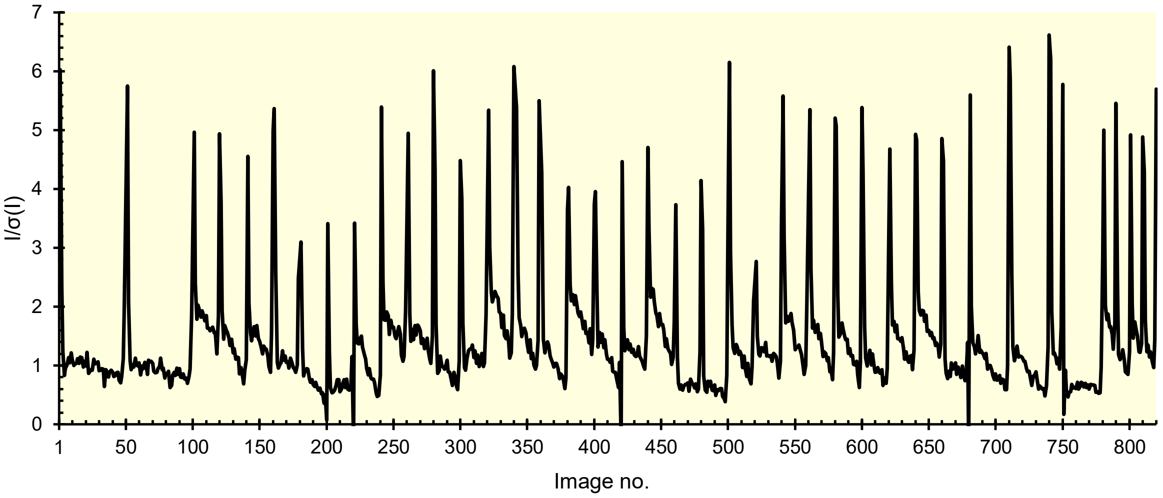

Figure S4. Representation of mean $I/\sigma(I)$ of reflections on a frame-by-frame basis concatenated for data collections of 39 crystals used in rotation-MCXRD data collections for **MOF-919(Sc/Cu)**. I/σ(I) per image values as reported from *xia2.multiplex*.

S5. Comparison of stationary-MCXRD (SX), rotation-MCXRD and SCXRD crystal structures of MOF-919(Sc/Cu)

Despite the radiation damage evident in the rotation-MCXRD data, these data provide an improvement in data quality and a decrease in radiation damage relative to the recently reported SCXRD data set.[S38] Our data illustrate that shorter exposure to reduce radiation damage could improve data quality even further, which can only be achieved by moving to stationary-MCXRD (SX) methods. This section expands upon the overview of X-ray induced changes given in the main text and illustrated in Figure 5a by providing a brief comparison between the crystal structure determinations using these three methods, each of which have endeavoured to get the maximum structural information from the data quality that can be obtained by a given approach.

The MOF-919(Sc/Cu) MOF has a framework chemical formula of $C_{24}H_{27}N_{12}O_{24}Cu_6Sc_3$, which arises from $[\{Sc_3(\mu_3\text{-}O)(OH)_3\}(\mu\text{-}PyC)_6\{Cu_3(\mu_3\text{-}O)(OH_2)_3\}_2]$, H_2PyC = 4-pyrazolecarboxylic acid. The crystal structure has Z = 272, but as the space group Fd-3m has 192 symmetry operations, the asymmetric unit of the crystal structure contains $1^5/_{12}$ of the $[\{Sc_3(\mu_3\text{-}O)(OH)_3\}(\mu\text{-}PyC)_6\{Cu_3(\mu_3\text{-}O)(OH_2)_3\}_2]$ units. Our models refined against the rotation-MCXRD or stationary-MCXRD data include all atoms in the formula, whereas the reported SCXRD study has a formula of $C_{22.76}H_0N_{11.64}O_{17.18}Cu_6Sc_3$ for the structure model used in the refinement, indicating some missing C, N, and O atoms and omission of all H atoms from the model. The refinement reported for the SCXRD study is highly constrained,[S38] using only 268 least-squares parameters and very few restraints (19) for a model comprising 105 independent atoms, whereas the models based on the MCXRD data were able to tolerate far greater flexibility of parameters in their refinement, with each study using 875 least-squares parameters alongside 1105 restraints for 120 independent non-hydrogen atoms and 45 hydrogen atoms.

Difference electron density maps calculated for the three crystal structures with all μ_3-O and OH_2 ligands of the Cu_3 SBUs omitted from the model are shown in Figure S5. Whereas electron density peaks indicating the presence of these oxide and water ligands are evident at the 2.0 e Å^{-3} contour level for the stationary-MCXRD (SX) data (Figure S5g), those for the rotation-MCXRD data are evident at 1.1 e Å^{-3} (Figure S5e) but the oxide and one of the three water molecules is not evident at the higher contour level

(Figure S5h). For the SCXRD structure, electron density peaks associated with the ligands are absent at 1.1 e Å^{-3} (Figure S5f) and even at the 0.9 e Å^{-3} contour level there is only a small electron density peak for the oxide and none that can be associated with water ligands (Figure S5c). The absence of the oxide and water ligands is consistent with radiation-induced reduction of the Cu sites (Cu(II) to Cu(I)), as discussed in the main manuscript. Deficiencies of the SCXRD model are further highlighted by residual electron density (positive and negative associated with the Cu sites), that is not present in the MCXRD studies, and the small residual electron density associated with the missing ring carbon atom (Figure S5c).

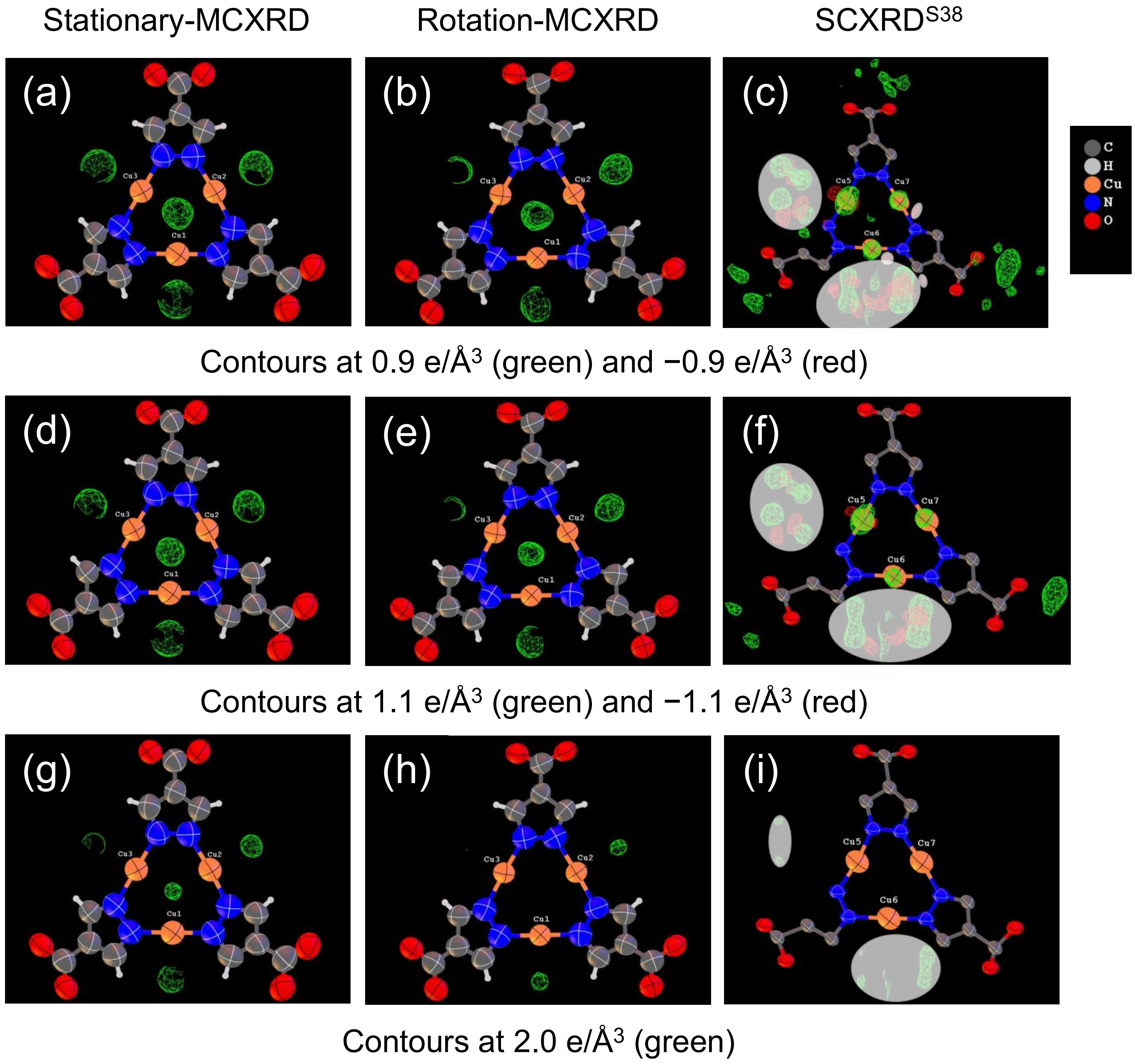

Figure S5. Residual electron density maps of $Cu_3(PyC)_3$ SBU comparing stationary-MCXRD (SX), rotation-MCXRD and SCXRD[S38] crystal structures of MOF-919(Sc/Cu), calculated with *OLEX2*. For all structures, any central μ_3-O and water ligands are not included in the model used to calculate the maps. Maps are presented with 3D contours shown at ±0.9 e Å^{-3}, ±1.1 e Å^{-3} and 2.0 e Å^{-3} as indicated. For the SCXRD study additional residual electron density peaks associated with other SBUs that lie well out of the plane of the $Cu_3(PyC)_3$ SBU shown have been partially obscured for clarity.

Displacement ellipsoid plots for the three crystal structure models are shown in Figure S6. Whereas restraints could be used to facilitate refinement of displacement parameters for the MCXRD models, all non-metal atoms required constrained displacement parameters in the SCXRD model.

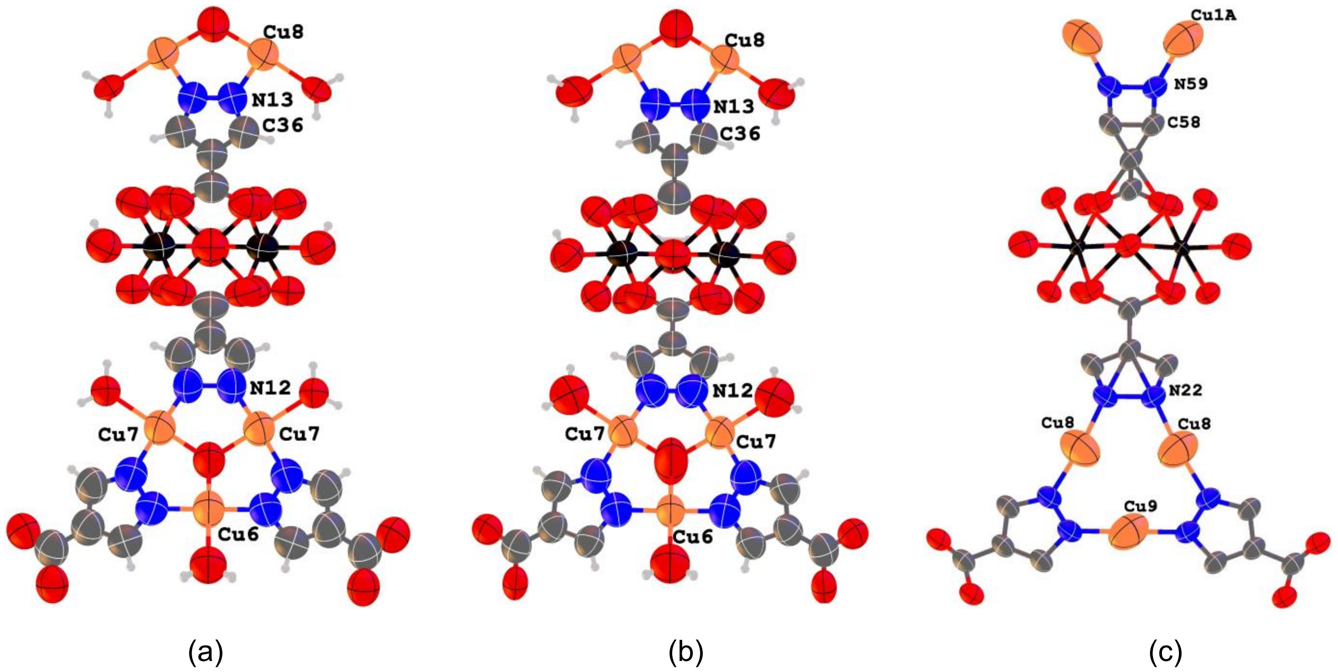

Figure S6. Ellipsoid plots (50% probability) comparing (a) stationary-MCXRD (SX), (b) rotation-MCXRD and (c) SCXRD[S38] crystal structures of MOF-919(Sc/Cu).

The aim of this comparison has not been to critique the SCXRD study, but rather to make clear that the study is undoubtably limited by severe radiation damage as a result of the large radiation dose that will have been needed to collect a full data set with a synchrotron X-ray source on one single crystal. Our MCXRD studies demonstrate that improvements in data quality for such materials can be achieved by spreading the total X-ray dose required across a few crystals many crystals (rotation-MCXRD) or even further by using a very large number of crystals (stationary-MCXRD, *i.e.* SX). This has enabled not only an improvement in the crystal structure model that can be achieved, but also the opportunity to study the material without (or at least without much of) the radiation-induced chemical changes.

S6. References

S1. Schaate, A.; Roy, P.; Godt, A.; Lippke, J.; Waltz, F.; Wiebcke, M.; Behrens, P. Modulated synthesis of Zr-based metal-organic frameworks: from nano to single crystals. *Chem. Eur. J.* **2011**, *17*, 6643−6651.

S2. Øien, S.; Wragg, D.; Reinsch, H.; Svelle, S.; Bordiga, S.; Lamberti, C.; Lillerud, K. P. Detailed Structure Analysis of Atomic Positions and Defects in Zirconium Metal−Organic Frameworks. *Cryst. Growth. Des.* **2014**, *14*, 5370–5372.

S3. Gándara, F.; Uribe-Romo, F. J.; Britt, D. K.; Furukawa, H.; Lei, L.; Cheng, R.; Duan, X.; O'Keeffe, M.; Yaghi, O. M. Porous, Conductive Metal-Triazolates and Their Structural Elucidation by the Charge-Flipping Method. *Chem. Eur. J.* **2012**, *18*, 10595–10601.

S4. Liu, Q.; Song, Y.; Ma, Y.; Zhou, Y.; Cong, H.; Wang, C.; Wu, J.; Hu, G.; O'Keefe, M.; Deng, H. Mesoporous Cages in Chemically Robust MOFs Created by a Large Number of Vertices with Reduced Connectivity. *J. Am. Chem. Soc.* **2019**, *141*, 488–496.

S5. Pangborn, A. B.; Giardello, M. A.; Grubbs, R. H.; Rosen, R. K.; Timmers, F. J.: Safe and Convenient Procedure for Solvent Purification. *Organometallics* **1996**, *15*, 1518-1520.

S6. This experimental set-up has been used previously for protein crystallization and X-ray data collection. Axford, D.; Aller, P.; Sanchez-Weatherby, J.; Sandy, J. Applications of thin-film sandwich crystallization platforms. *Acta Crystallogr.* **2016**, F*72*, 313–319.

S7. Grassucci, R. A.; Taylor, D. J.; Frank, J. Preparation of macromolecular complexes for cryo-electron microscopy. *Nat. Protoc.* **2007**, *2*, 3239–3246.

S8. Crawshaw, A. D.; Beale, E. V.; Warren, A. J.; Stallwood, A.; Duller, G.; Trincao, J.; Evans, G. A. Sample Preparation Pipeline for Microcrystals at the VMXm Beamline. *J. Vis. Exp.* **2021**, *172*, e62306.

S9. Winter, G. xia2: an expert system for macromolecular crystallography data reduction. *J. Appl. Cryst.* **2010**, *43*, 186–190.

S10. Gildea, R. J.; Beilsten-Edmands, J.; Axford, D.; Horrell, S.; Aller, P.; Sandy, J.; Sanchez-Weatherby, J.; Owen, C. D.; Lukacik, P.; Strain-Damerell, C.; Owen R. L.; Walsh, M. A.; Winter, G. *xia2.multiplex*: a multi-crystal data-analysis pipeline. *Acta Crystallogr.* **2022**, D*78*, 752–769.

S11. Winter, G.; Waterman, D. G.; Parkhurst, J. M.; Brewster, A. S.; Gildea, R. J.; Gerstel, M.; Fuentes-Montero, L.; Vollmar, M.; Michels-Clark, T.; Young, I. D.; Sauter, N. K.; Evans, G.; DIALS: implementation and evaluation of a new integration package. *Acta Crystallogr.* **2018**, D*74*, 85–97.

S12. Winter, G.; Beilsten-Edmands, J.; Devenish, N.; Gerstel, M.; Gildea, R. J.; McDonagh, D.; Pascal, E.; Waterman, D. G.; Williams, B. H.; Evans, G. DIALS as a toolkit. *Protein Sci.* **2022**, *31*, 232–250.

S13. Assmann, G.; Brehm, W.; Diederichs, K. Identification of rogue datasets in serial crystallography. *J. Appl. Cryst.* **2016**, *49*, 1021–1028.

S14. Karplus, P. A.; Diederichs, K. Linking crystallographic model and data quality. *Science* **2012**, *336*, 1030–1033.

S15. Weiss, M. S. Global indicators of X-ray data quality. *J. Appl. Cryst.* **2001**, *34*, 130–135.

S16. Weiss, M. S.; Hilgenfeld, R. On the use of the merging R factor as a quality indicator for X-ray data. *J. Appl. Cryst.* **1997**, *30*, 203–205.

S17. Beilsten-Edmands, J.; Parkhurst, J. M.; Winter, G.; Evans, G. Processing serial synchrotron crystallography diffraction data with DIALS. *Methods Enzymol.* **2024**, *709*, 207-244.

S18. Sheldrick, G. M. A short history of *SHELX*. *Acta Crystallogr.* **2008**, A*64*, 112–122.

S19. Sheldrick, G. M. SHELXT – Integrated space-group and crystal-structure determination. *Acta Crystallogr.* **2015**, A*71*, 3–8.

S20. Sheldrick, G. M. Crystal structure refinement with SHELXL, *Acta Crystallogr.* **2015**, C*71*, 3–8.

S21. Dolomanov, O. V.; Bourhis, L. J.; Gildea, R. J.; Howard, J. A. K.; Puschmann, H. *OLEX2*: a complete structure solution, refinement and analysis program. *J. Appl. Cryst.* **2009**, *42*, 339–341.

S22.The *BYPASS* solvent mask in *OLEX2* is based upon: Jiang, J.-S.; Brunger, A. T. Protein Hydration Observed by X-ray Diffraction: Solvation Properties of Penicillopepsin and Neuraminidase Crystal Structures. *J. Molec. Biol.* **1994**, *243*, 100–115.

S23. Evans, G., Alianelli, L., Burt, M., Wagner, A. & Sawhney, K. J. S. Diamond Beamline 124: A Flexible Instrument for Macromolecular Micro-crystallography, in *Synchrotron Radiation Instrumentation*; Choi, J. Y.; Rah, S., Eds.; American Institute of Physics: New York, **2007**; pp. 836–839.

S24. Feng, D.; Wang, K.; Wei, Z.; Chen, Y.-P.; Simon, C. M.; Arvapally, R. K.; Martin, R. L.; Bosch, M.; Liu, T.-F.; Fordham, S.; Yuan, D.; Omary, M. A.; Haranczyk, M.; Smit, B. Zhou, H.-C. Kinetically tuned dimensional augmentation as a versatile synthetic route towards robust metal–organic frameworks. *Nature Comm.* **2014**, *5*, No. 5723.

S25. Guzei, I. A. An idealized molecular geometry library for refinement of poorly behaved molecular fragments with constraints. *J. Appl. Cryst.* **2014**, *47*, 806-809.

S26. Dominic Bara, Ph.D. Thesis, University of Glasgow, 2020.

S27. Serre, C.; Mellot-Draznieks, C.; Surblé, S.; Audebrand, N.; Filinchuk, Y.; Férey, G. Role of Solvent-Host Interactions That Lead to Very Large Swelling of Hybrid Frameworks. *Science,* **2007**, *315*, 1828-1831.

S28. Pawley G. S., Unit-cell refinement from powder diffraction scans *J. Appl. Cryst.* **1981**, *14*, 357–361.

S29. Duke, E. M. H.; Evans, G.; Flaig, R.; Hall, D. R.; Latchem, M.; McAuley, K. E.; Sandy, D. J.; Sorensen, T. L-M.; Waterman, D.; Johnson, L. N. The Phase I MX Beamlines at Diamond Light Source. *AIP Conf. Proc.* **2010**, *1234*, 165–168.

S30. Smith, R.; Brammer, L. Transformations of Aromatic Alcohols Catalysed by a Molybdate-Modified Metal-Organic Framework, Abstract B3.05, MOF2016, Long Beach, CA, USA, Sept. 2016.

S31. Noh, H.; Kung, C.-W.; Otake, K.; Peters, A. W.; Li, Z.; Liao, Y.; Gong, X.; Farha, O. K.; Hupp, J. T. Redox-Mediator-Assisted Electrocatalytic Hydrogen Evolution from Water by a Molybdenum Sulfide-Functionalized Metal−Organic Framework. *ACS Catal.* **2018**, *8*, 9848–9858.

S32. Warren, A. J.; Trincao, J.; Crawshaw, A. D.; Beale, E. V.; Duller, G.; Stallwood, A.; Lunnon, M.; Littlewood, R.; Prescott, A.; Foster, A.; Smith, N.; Rehm, G.; Gayadeen, S.; Bloomer, C.; Alianelli, L.; Laundy, D.; Sutter, J.; Cahilla, L.; Evans, G. VMXm – A sub-micron focus macromolecular crystallography beamline at Diamond Light Source. *J. Synchrotron Rad.* **2024**, *31*, 1593–1608.

S33. The initial report of the crystal structure of MOF-919(Sc/Cu) modelled from PXRD data by Rietveld refinement has coordinates available via the CCDC deposition system (https://www.ccdc.cam.ac.uk/structures/) as deposition number 1873016. Its entry in the Cambridge Structural Database (CSD) as refcode DIMBUZ does not have retained atomic coordinates.

S34. Zeldin O. B.; Gerstel M.; Garman E. F. RADDOSE-3D: Time- and space-resolved modelling of dose in macromolecular crystallography. *J. Appl. Cryst.* **2013**, *46*, 1225–1230.

S35. Garman Group Github page *RADDOSE-3D* manual, https://github.com/GarmanGroup/RADDOSE-3D/blob/master/doc/user-guide.pdf (accessed September 2024).

S36. Owen, R. L.; Holton, J. M.; Schulze-Briese, C.; Garman, E. F. Determination of X-ray flux using silicon pin diodes. *J. Synchrotron Rad.* **2009**, *16*, 143–151.

S37. Zeldin, O. B.; Brockhauser, S.; Bremridge, J.; Holton, J. M.; Garman E. F. Predicting the X-ray lifetime of protein crystals. *Proc. Natl. Acad. Sci.*, *USA* **2013**, *110*, 20551–20556.

S38. Hu, G.; Liu, Q.; Zhou, Y.; Yan, W.; Sun, Y.; Peng, S.; Zhao, C.; Zhou, X.; Deng, H. Extremely Large 3D Cages in Metal−Organic Frameworks for Nucleic Acid Extraction. *J. Am. Chem. Soc.* **2023**, *145*, 13181−13194.
